# Influence of motor skills training on children’s development evaluated in the Motor skills in PreSchool (MiPS) study-DK: study protocol for a randomized controlled trial, nested in a cohort study

**DOI:** 10.1186/s13063-017-2143-9

**Published:** 2017-08-29

**Authors:** Lise Hestbaek, Sarah Thurøe Andersen, Thomas Skovgaard, Line Groenholt Olesen, Mette Elmose, Dorthe Bleses, Simon Calmar Andersen, Henrik Hein Lauridsen

**Affiliations:** 1 Nordic Institute of Chiropractic and Clinical Biomechanics and Department of Sports Science and Clinical Biomechanics, University of Southern Denmark, Campusvej 55, 5230 Odense M, Denmark; 2 Department of Sports Science and Clinical Biomechanics, University of Southern Denmark, Campusvej 55, 5230 Odense M, Denmark; 3 Institute of Psychology, University of Southern Denmark, Campusvej 55, 5230 Odense M, Denmark; 4 TrygFonden’s Centre for Child Research, Department of Economics and Business Economics, and School of Communication and Culture, Aarhus University, Aarhus, Denmark; 5 TrygFonden’s Centre for Child Research, Department of Economics and Business Economics, Aarhus University, Aarhus, Denmark

**Keywords:** Children, Motor skills, Preschool, Kindergarten, Physical activity, Musculoskeletal, Wellbeing, Cognitive development

## Abstract

**Background:**

Good motor skills are considered important for children’s physical, social, and psychological development, but the relationship is still poorly understood. Preschool age seems to be decisive for the development of motor skills and probably the most promising time-window in relation to preventive strategies based on improved motor skills. This research program has four overall aims: (1) investigation of the effect of a structured program aimed at improving motor skills in 3–6-year-old children on current and future motor skills, health, cognition, and wellbeing; (2) establish reference data on motor skills in 3–6-year-olds; (3) description of early development of musculoskeletal problems; and (4) establishment of a population-based cohort of 3–6-year-olds.

**Methods:**

Over a four-year period, all preschools in a Danish municipality, Svendborg, will implement a new program aimed at optimizing children’s motor skills. By introducing the program into a subset of the preschools at onset and comparing these children to another subset (control) that will not receive the intervention the first three years, it is possible to document a potential effect of the intervention. At the same time, a cohort will be established including all children attending preschools in the municipality with extensive baseline data collection: gross and fine motor skills; movement patterns; musculoskeletal complaints; physical activity; anthropometry; general wellbeing; cognitive abilities; language status; medical history; demographic background; and more.

The children are aged 3–6 years at baseline. A total of 1461 children have been invited into the cohort, 368 to the intervention arm and 359 to the control arm. Follow-up time for the trial is 2.5 years. The cohort is planned to run at least until the children leave school at age 15–16 years. Longer follow-up will depend on future funding.

**Discussion:**

If the results of the trial are positive, the intervention can be implemented in other similar settings with reasonable ease and at a relatively low initial cost. This is due to the extensive end-user involvement, the broad population base, and the pragmatic nature of the intervention. The cohort will provide important information about the influence of early motor skills on children’s development across many domains and the potential interactions between these domains.

**Trial registration:**

ISRCTN registry, ISRCTN23701994. Registered on 13 October 2016.

**Electronic supplementary material:**

The online version of this article (doi:10.1186/s13063-017-2143-9) contains supplementary material, which is available to authorized users.

## Background

In recent years, Danish preschools have had an increasingly strong focus on improving children’s motor skills. Several projects have been implemented both by municipalities and by larger sports organizations. For example, Sport Confederation Denmark has developed a program to improve motor skills in preschool children in cooperation with the YMCA and this is currently being offered to more than 40,000 children across Denmark (http://www.rendoghop.info/). Therefore, it seems timely to initiate scientific investigations into the effectiveness and potential benefits of such programs.

Good motor skills are considered important for children’s physical, social, and psychological development [1] and may even be the foundation for an active lifestyle, since several studies have shown a positive association between good motor skills and higher levels of physical activity [2–4]. Consequently, there is evidence of many health benefits to be gained from an improvement in motor skills. For instance, it has been demonstrated that good motor skills positively influence cardiorespiratory fitness [2, 5] and body weight [2, 6–8] as well as sports participation [2, 9], all suggesting that early competency in motor skills may have important health implications. Furthermore, there are indications of relationships with language development [10–14], executive function [15], and general wellbeing [16]. However, most of the existing studies of motor performance are either cross-sectional, and therefore do not provide evidence of a potential causal relationship, or they include only short-term follow-up. Thus, there is a real need for more longitudinal studies on the importance of motor skills with long-term follow-up, including both physical and psychological outcomes.

There are indications that the level of motor skills remains stable over time [17] and motor development deficits observed in early childhood are still apparent in adolescence [18]. Therefore, toddler and preschool age appears to be a particularly important period for the development of motor skills. Early childhood is also the age where practicing fundamental movement skills is necessary to create a foundation for more complex movement activities of daily living, recreation, and sports in later childhood [1]. In Denmark, 92% of all 3–5-year-old children spend a high proportion of their waking hours at preschool [19]. Consequently, this arena provides an ideal opportunity for all children, despite socioeconomic background, to develop and improve their motor skills.

The Municipality of Svendborg has previously conducted the CHAMPS-DK study (The Svendborg Project) [20] in cooperation with the University of Southern Denmark, which was a field experiment investigating the effect of more physical education in primary schools. This project was concluded in 2014 and, among other findings, demonstrated that more physical education in school improved bone development [21], reduced cardiovascular risk [22], reduced the prevalence of overweight [23], and improved physical fitness in those with a low baseline level [24]. As a result, more physical education lessons have been added to the schedule of all schools within the Municipality. The Municipality is comparable to the rest of Denmark in terms of age distribution, gender, and income, but with a slightly higher unemployment rate (5.3% vs. 4.5%) [19]; therefore, the results will be transferable to the rest of the country.

Understanding the importance, but also the challenge, of early prevention, the Municipality is now ready to continue the evidence-based improvement of children’s lives and has initiated an intervention in its preschools aimed at improving motor skills in children. Once more, the Municipality is focused on increasing the evidence base through scientific investigation and has proposed a partnership where the Department of Sports Science and Clinical Biomechanics (DSSCB) at the University of Southern Denmark (SDU) develop and manage the scientific design and evaluation of the project – creating the unique research opportunities outlined in this protocol.

The effectiveness of the intervention will be investigated in a cluster randomized controlled trial (RCT) focused on improvement in motor skills as well as several secondary effects. Importantly, the extensive testing of these children at an early age will form the basis of a cohort with potential for long-term follow-up, which will enable investigations into the long-term development of motor skills, musculoskeletal disorders, physical activity, language, cognitive abilities, and social skills as well as the interrelations between these domains. In addition, the predictive ability of early markers for child development and health within these domains can be assessed. Thus, in addition to assessing the effectiveness of the intervention developed for this project, we will provide an evidence base for future strategies for optimizing children’s health, wellbeing, and cognitive and language development. Research plans for each of these individual domains are described in separate work packages (WPs) in Additional file 1.

### Aims and objectives

The four overall aims of this research program are to: (1) establish a population-based cohort of 3–6-year-olds with a focus on the relationship between motor skills, health, cognition, and wellbeing; (2) establish population-based reference data on motor skills in 3–6-year-olds; (3) describe early development of musculoskeletal problems; and (4) investigate whether a structured program aimed at improving motor skills in 3–6-year-old children will improve current and future motor skills, health, cognition, and wellbeing

## Methods

Since this study includes both a cohort study and a RCT nested within that cohort, the method section is divided into two sections for clarity. The cohort will be described first, followed by the RCT.

Furthermore, a total of eight WPs have been developed representing different domains. The WPs are designed to use data from both the cohort and the RCT, including:

- WP 1: Effectiveness of a structured intervention for improving motor skills in Danish preschool children
- WP 2: Process evaluation
- WP 3: Motor performance and musculoskeletal disorders
- WP 4: Motor skills influence on physical activity and overweight; and population-based motor skills reference data
- WP 5: Associations between early language and motor skills
- WP 6: Association between motor skills and wellbeing
- WP 7: Motor skills and effects on executive functions
- WP 8: Associations between motor skills, physical activity, cognition, psychological wellbeing, and language development for children at developmental risk

All the WPs are described in detail in Additional file 1 and this main protocol describes the general procedures creating the basis for the WPs.

### Cohort study

#### Objectives to be investigated using a cross-sectional and longitudinal cohort design

- Establish normative Danish data on motor skills at ages three, four, and five years
- Determine incidence, prevalence, and patterns of development of musculoskeletal problems in children
- Study the relationship between motor skills and musculoskeletal health
- Study the relationship between motor skills, physical activity, and overweight
- Study the relationship between motor skills and language development
- Study the relationship between motor skills and wellbeing
- Study the relationship between motor skills and executive function

More detailed objectives are described in the WPs (Additional file 1).

### Design

A preschool-based cohort study with 12 years of follow-up.

### Participants

All the children attending public preschools in the Municipality of Svendborg (84% of the population in the age group) were invited to participate in the study. In August 2016, this involved 1461 children attending 32 preschools. In Denmark, children start preschool when they turn three years of age and leave preschool in July of the calendar year they turn six years, i.e. the sample were born in 2011, 2012, or 2013. All parents received written information about the project and were invited to information meetings at local schools or preschools during the spring of 2016. Written consent forms were submitted to the children’s respective preschools before September, 2016. Following the initial inclusion, there is running inclusion into the study. By December 19, 2016, the parents of 865 children (59%) had agreed to participate in the study

### Eligibility criteria

All children attending the municipal preschools were eligible, pending parental consent.

### Data collection methods

Trained research staff will collect data from physical testing during test sessions. These test sessions will take place in gyms in the proximity of the preschools. The children will either walk or be transported in buses, depending on the distance, and they will be accompanied by their known preschool staff. Other data will be collected via interviews, email surveys, or SMS-track as described in the individual WPs (Additional file 1). An overview of the investigated domains and their related measurements is presented in Table 1.

**Table 1:** Overview of the investigated domains

Domain	Focus areas	Measured by^a^
Motor skills (WP1)	Gross and fine motor skills	-Movement Assessment Battery for Children -2 (M-ABC2)
	Physical competence	-Handgrip strength, jump length, 20 m run
	Self-perceived motor performance	-Perceived physical subscale of PSPSCA
		-Parent-perceived motor skill competence
Implementation (WP2)	Reach	-The target population is compared to the larger “denominator” population
	Efficacy	-CF. Information on *Variables* and *Analyses of effect* for WP1
	Adoption	-The target staff and institutions are compared to the larger “denominator” population
Musculoskeletal (MSK) health (WP3)	Number of MSK complaints	-SMS questions to parents
	Detection of MSK complaints	
	Movement patterns	-The Captury System (3D motion capture)
	Diagnoses	-Examination by orthopedic surgeon
Physical activity and anthropometry (WP4)	BMI	-Height and weight
	Waist/height ratio	-Measured in cm and ratio calculated
	Physical activity	-Accelerometers
Language (WP5)	Oral language skills	-Language assessment 3–6 administered by preschool staff
	Literacy skills	
Wellbeing (WP6)	Psychological wellbeing	-SDQ – Parent and staff
	Physical and emotional wellbeing	-KINDLR – Parent
	Self-esteem	
	General wellbeing	-KINDLR – Parent
		- SDQ – Parent, staff, and child (age 11 years and older)
	Social behavior	-KINDLR – Parent and child (age 7 years and older)
	Social competencies	-KINDLR – Parent and child (age 7 years and older)
Cognition (WP7)	Self-regulation	-The Preschool Self-Regulation Assessment Battery (PSRA)
	Estimated general intelligence	-Reynold’s intellectual Assessment Screening Tool (RIST)
		-School grades
	Academic performance	-National tests
Developmental difficulties (WP8)	Formal diagnosis	-SDQ algorithms for likelihood of psychiatric diagnoses
		-Formal psychiatric diagnoses from registries

^a^Full descriptions in the respective WPs

*WP* work package

### Variables

#### Core variables for all WPs

Motor skills, physical fitness and functional performance, and anthropometry will be measured by trained research staff at baseline and after 6, 18, and 30 months, as long as the child attends preschool. Furthermore, all parents will be asked to fill out a baseline questionnaire with information about socioeconomic status, their own and their children’s health, physical activity, IT and reading habits, etc.

For long-term follow-up, anthropometry will be measured regularly by the school nurse; academic examination grades, results from national academic tests as well as national tests of general wellbeing during the school years will be available. If funding is procured, additional physical testing will be performed during the school years.

##### Motor skills

Motor skills will be tested with the revised version of the Movement Assessment Battery for Children (MABC-2). This test battery assesses the developmental status of fundamental movement skills in children and includes eight individual test items measuring movement skills in three categories: manual dexterity skills; ball skills; and balance skills. Each item is rated on a six-point rating scale, where 0 indicates the best performance and 5 the weakest. A total impairment score expresses the child’s test performance and profile scores provide information about the child’s performance of each individual category [25].

The revised version of the test is subdivided into three age bands (3–6, 7–10, and 11–16 years) [26]; thus, the first age band will be used during the preschool years in this project. The revised version also has qualitative assessment added, but only the quantitative assessments will be used in this study.

The test (both the original and the revised) has been validated in several countries [27–31] and translated into several languages, including Danish, and is widely used in Denmark [32]. The cross-cultural validation, the availability in several European countries, and the simple test administration, facilitating large sample screening over a short period, are considered major advantages of this test [33].

##### Physical fitness and functional performance

Physical fitness and lower extremity functional performance will be tested by measuring two gross motor skills: the time taken to complete a 20-m fast run and the distance of a horizontal jump (standing long jump). The fast run will be measured in seconds and the horizontal jump in centimeters.

Handgrip strength using the non-digital version (model 5001) of the TKK dynamometer (0–100 kg) will be used to assess strength and functional performance of the upper extremity. Grip strength will be measured in kilograms.

##### Anthropometry

Height, weight, and waist circumference will be measured using standard anthropometric methods with the children wearing normal light clothes. Body mass index (BMI), waist circumference, and waist to height ratio will be calculated as indicators of general and abdominal adiposity and are valid measures of adiposity in preschool children [34].

##### Other variables

Several other variables will be collected for the different WPs. An overview is provided in Table 1 and more detailed descriptions in Additional file 1.

### Analyses

Simple univariate statistics will be used to describe the outcomes, predictors, and covariates. Age-specific, sex-specific, and age- and sex-specific rates of the various outcomes will be presented.

In most instances, multilevel regression analyses will be used to determine if poor motor skills are associated with other domains after adjusting for relevant covariates. The choice of regression type, covariates and interactions is described in each WP in Additional file 1.

Longitudinal latent class analysis and/or latent class growth analysis will be conducted to describe developmental patterns.

### Randomized controlled trial

Objectives to be investigated using a cluster RCT design are:

- Can movement patterns be improved through a structured motor skills intervention in preschool?
- Can physical activity be increased through a structured motor skills intervention in preschool?
- Can language development be improved through a structured motor skills intervention in preschool?
- Can wellbeing be improved through a structured motor skills intervention in preschool?
- Can executive function be improved through a structured motor skills intervention in preschool?
- How does a universally implemented motor skills intervention affect children with developmental difficulties?
- Evaluation of the implementation success of a motor performance intervention in preschools.

More detailed objectives are described in the WPs (Additional file 1).

### Design

A field experiment with a cluster RCT nested in the cohort study described above.

### Participants

Children from all the 32 preschools in the municipality were included in the cohort study. The governing boards of 17 of these, representing 834 children by August 2016, agreed to be included in the RCT. Only children enrolled in the cohort before the end of January 2017 will be included in the RCT, as that represents the end of the baseline data collection. By December 19, 2016, 485 children were included in the RCT.

### Eligibility criteria

The eligibility criteria are the same as for the cohort study.

### Randomization

Participating preschools were randomized, stratified for socioeconomic background. A mean socioeconomic index for each preschool was developed based on family type, education, and income, and this was dichotomized to above or below the median for all the included kindergartens. This was done by a statistician at the SDU.

The randomization resulted in eight preschools, including a total of 368 children, in the intervention group and nine preschools, including a total of 359 children, in the control group.

### Sample size calculation

We are not aware of any previous studies using the same test in the same age groups and therefore a power calculation for the RCT was performed using data from related studies. The standard deviations of the change in motor skills was based on a study of Danish overweight children [32], which used the same motor assessment as in this study (Movement Assessment Battery for Children), and the clustering effect was estimated based on a previous study in Danish preschools [35].

Based on the recommendations by In [36], the effect size was expressed as standard deviations units, since exact prior knowledge is not available. He states: “When prior knowledge for the calculation of the standardized effect size is not sufficient, a commonly applied effect size is 0.25–0.50, which was initially suggested by Cohen and which is still important” [36]. Hence, we intended to recruit a sample large enough to detect a reduction in the standard deviation score of 0.30 with a power of 80% at a significance level of 5%. Correlation within preschools and hence the loss of efficiency because of clustering was taken into account by adjusting for intraclass correlation (ICC) in the primary sampling units (ICC = 0.015). This correlation was based on our previous, but unpublished, experience in a similar setting of preschools in Denmark. This power calculation resulted in a required number of 468 participants. Such a calculation should be considered with caution since the underlying estimates of variance and cluster effects are not directly transferable. However, the population size seems sensible in comparison to other trials investigating motor activities in preschools. Piek et al. included almost the same, 501 children [37], whereas Roth et al. investigated the effect of a physical activity intervention in preschools in a somewhat larger sample of 709 children [38]. However, many studies have demonstrated statistically significant effects with considerably smaller samples [39–42].

By December 19, 2016, 485 children were included in the RCT (67% of eligible children).

### Data collection methods

Part of the data collection for the cohort described above.

### Intervention

The intervention has been developed by a working group that includes representatives from the research team, the participating preschools, the Municipality, and independent experts, in an iterative process aimed at ensuring “buy-in” and “ownership” of all stakeholders. The focus is on movement, development of motor skills, and body awareness. The intervention will comprise gross motor challenges, fine motor challenges, coordination exercises, balance exercises, challenging of the vestibular, tactile and kinaesthetic senses, and relaxation. The intervention is described in detail in Additional file 2b.

Through the collaborative partnership with the individual preschool institutions, the intervention is designed to be flexible and adaptive to ease implementation. Importantly, qualitative and quantitative minimum requirements have been defined and these will be monitored as part of the process evaluation (WP2).

Researchers and independent experts in the competency development group will provide examples and targets for motor skills, whereas preschool staff will modify the intervention strategies to suit their physical environment, culture, and daily schedule. Hence, the intervention is not a strictly defined curriculum, but a strategy to enhance motor skills during the preschool day while fulfilling the minimum criteria.

#### Implementation

Because the concept does not foresee implementation control on a day-to-day basis, a number of initiatives are launched that are designed to support the effective implementation of the concept.

- Network of coordinators
- Regular written evaluations
- Ownership and flexibility

To support the implementation of the concept, a network of coordinators will be established from the participating children’s institutions. Network meetings will be held where coordinators regularly explain and discuss the implementation in their respective institutions. This offers the opportunity to solve any challenges along the way and provides mutual inspiration. Experience from the sports school project shows that such a network is a good tool to support implementation and overcome the challenges that may arise during implementation. At the same time, the coordinators and managers will maintain ongoing evaluation schemes, aiming to uncover implementation and possible challenges, which can then be addressed.

To support the overall work on the concept, the staff groups in the individual preschools draw and describe the specific plans for integrating the requirements of the concept within the structure of the given institutions, both in relation to the physical surroundings and their daily practices. This provides flexibility and ownership in individual preschools, making it more likely that the implementation of the individual houses will succeed.

#### Competency development

Svendborg Municipality recognizes that the requirements for effective integration of the overall motor skills program into the institutions' daily practice are considerable and that not all staff members can contribute off hand. Therefore, funds have been allocated to the preparation of a competency development program, supplying enrolled staff with tailored knowledge, skills, and capacity to deliver the program. At the same time, resources for 37 h of training for all preschool staff in the participating preschools have been allocated.

As has been the case with the other working groups, both preschool leaders and staff are represented in the working group for competency development, along with a physiotherapist from the pedagogical and psychological advisory unit, a healthcare consultant, and others. Additionally, cooperation with the regional University College has been established.

### Control group

In the control group, there is no interference from either the research group or from the municipality. The preschools in this group simply continue their usual practices, with the only exception being the testing of the children by the research team.

### Variables

The variables are the same as for the cohort described above.

### Analyses

The primary outcome will be motor skills as measured by the MABC-2 at the 18-month follow-up (Spring 2018). At this point, the children will have received the intervention for 12 months after a six-month phase-in period.

Multilevel linear regression models will be constructed to assess the effectiveness of the intervention on motor skill performance. The baseline value of the outcome variable, time since baseline, intervention group, and any imbalanced covariates will be added to the model along with preschool and socioeconomic status of the area as random effects. The interaction between preschool and intervention group will be tested. More details are provided in Additional file 1.

Secondary analyses will investigate the effect of the intervention on other domains using different types of regression analyses, depending on the outcome. Specific strategies for the specific research questions, as well as choice of covariates and included interactions, are described in Additional file 1.

### Administrative information

**Fig. 1:**
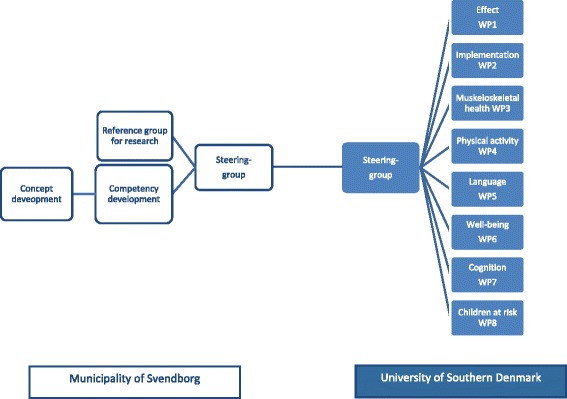
The MiPS study-DK. Organizational diagram

Roles and responsibilities are:

1. *Organization within the Municipality of Svendborg*
  The project structure within the Municipality of Svendborg is described in Additional file 2. In brief, it consists of a steering group including Lise Hestbaek as representative of the University of Southern Denmark (SDU), a workgroup for “Concept development” including Lisbeth Runge Larsen as representative of SDU, a workgroup for “Competency development,” and a “Reference group for research” including Lise Hestbaek and Henrik Hein Lauridsen as representatives of SDU. Names and affiliations of all involved can be seen in Additional file 2a.
2. *Organization within the SDU*
  2.1 *Steering group:* During the planning process (December 2015–September 2016), the steering group met three times to ensure progression in the practical aspects of the project and to discuss and contribute to scientific issues. From September 2016 to June 2019, the group will meet every six months or more frequently if needed. After June 2019, the group will meet as required. The steering group will be responsible for granting access to data.
    Lise Hestbaek – Associate Professor, Research Unit for Clinical Biomechanics, DSSCB, SDU. Senior Researcher, Nordic Institute of Chiropractic and Clinical Biomechanics, Odense. *Chair*
    Birgit Lindbergh – Head of Secretariat and Daycare, Municipality of Svendborg
    Jan Hartvigsen – Professor, Head of Research Unit for Clinical Biomechanics, DSSCB, SDU
    Peter Lund Kristensen – Associate Professor, Research Unit for Exercise Epidemiology and the center for Research in Childhood Health, DSSCB, SDU
    Dorthe Bleses – Professor, The Tryg Foundation’s Centre for Child Research Department of Economics and Business Economics and School of Communication and Culture, University of Aarhus
    Mette Elmose Andersen – Assistant professor, Institute of Psychology, SDU
    Jens Troelsen – Professor, Head of Research Unit “Active Living,” DSSCB, SDU
  2.2 *Research group, individuals responsible for the specific WPs:* During the pilot phase and the baseline data collection (April 2016–January 2017), the research group will meet every other month to coordinate the project. From January 2017 to June 2019, the group will meet as needed, but at least every three months.
    The organization is illustrated in Fig. 1.

    WP 1: Lise Hestbaek, Associate Professor, Research Unit for Clinical Biomechanics, DSSCB, SDU. Senior researcher, Nordic Institute of Chiropractic and Clinical Biomechanics, Odense
    WP 2: Jens Troelsen, Professor, Head of Research Unit “Active Living,” DSSCB, SDU; and Associate Professor Thomas Skovgaard, Research Unit Active Living and Head of “Research and Innovation Center for Human Movement and Learning,” DSSCB, SDU/UCL
    WP 3: Henrik H. Lauridsen, Associate Professor, Research Unit for Clinical Biomechanics, DSSCB, SDU
    WP 4: Peter Lund Kristensen, Associate Professor, Research Unit for Exercise Epidemiology and Research in Childhood Health, DSSCB, SDU; and Anders Grøntved – Associate Professor, Head of Research Unit for Exercise Epidemiology and the Centre “Research In Childhood Health,” DSSCB, SDU
    WP 5: Dorthe Bleses, Professor, The Tryg Foundation’s Centre for Child Research Department of Economics and Business Economics and School of Communication and Culture, University of Aarhus
    WP 6: Mette Elmose, Assistant Professor, Department of Psychology, SDU
    WP 7: Simon Calmar Andersen, Professor, TrygFonden’s Centre for Child Research, Department of Economics and Business Economics, Aarhus University
    WP 8: Mette Elmose, Assistant Professor, Department of Psychology, SDU

### Timeline

**Fig. 2:**
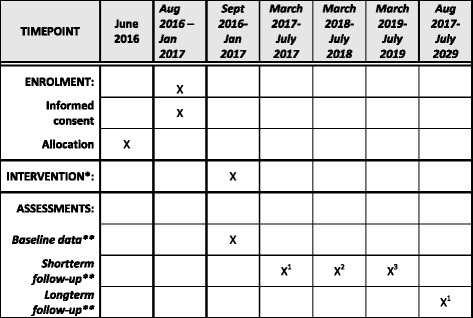
SPIRIT diagram for the schedule of enrolment, interventions, and assessments. *See description of the intervention in main protocol and Additional file 2. **See description of collected variables in main protocol and Additional file 1. ^1^ includes all the children, i.e. children born in 2011, 2012, or 2013; ^2^ includes children born in 2012 or 2013, since children from 2011 have left preschool; ^3^ includes children born in 2013, since children born in 2011 and 2012 have left preschool

The intervention is gradually introduced from September 2016 to January 2017 and baseline measures are obtained in the same period. The core variables described above will be measured 6, 18, and 30 months after baseline for the full cohort as long as they attend preschool. This means that children born in 2011 will have this type of follow-up once (Spring 2017), children born in 2012 twice (Spring 2017 and Spring 2018), and children born in 2013 three times (Spring 2017, Spring 2018, and Spring 2019). Other variables will be collected as described in the respective work packages. The timeline and sample is summarized in the SPIRIT diagram in Fig. 2.

### Data handling

#### Data management and monitoring

A data manager has been employed to oversee recording, cleaning, storage, and extraction of data. Questionnaire and most test data will be administered and stored by the Odense Patient data Explorative Network (OPEN), which is a research infrastructure facility based at Odense University Hospital. Films, and other data from the kinematic evaluations (WP3) will be stored on a secure server at the SDU. The RIST test (WP7) will be recorded on paper and stored on the same server as the kinematic data. Both OPEN and the data manager will monitor data continuously and therefore a data monitoring committee is redundant. After conclusion of the project, data will be transferred to the Danish National Archives.

#### Harms and auditing

Since the intervention simply focuses on activities usually performed by this age group, and the research solely documents effects of an intervention which would take place regardless, auditing is not relevant and discontinuation of the intervention is up the administration in the municipality and not a research decision. However, there will be continuous recording of musculoskeletal problems to document a potential risk of injury related to the intervention (WP3).

### Dissemination

Knowledge and insights gained through this research will be disseminated to scientific communities, preschool staff, primary care clinicians, opinion- and decision-makers, and finally to the public, using a variety of avenues.

Scientific publications will be published in international peer-reviewed journals for both short-term and long-term results of the WPs outlined in this application. Results will also be presented at international scientific conferences.

An important target for dissemination is preschool staffs which are in daily contact with most of the children aged 3–6 years in Denmark. The intervention will be implementable in mainstream preschools all over the country and the principles, requirements, and benefits will be communicated through professional journals and relevant web sites to preschool teachers, leaders, administrators, and decision-makers. Primary care clinicians will be informed of the results of the studies via articles in professional journals such as the *Journal of the Danish Medical Association*, *The Physiotherapist*, and *The Chiropractor* so they are aware of the benefits of good motor skills. This is likely to result in continuing educational efforts through the university colleges and other relevant educational providers, starting with University College Lillebaelt (UCL), which is also part of the concept and competency development groups.

Communication through the media and popular press will be guided by the press office at SDU and through close collaboration with the administration in the Svendborg Municipality (The Department of Children and Adolescents and the Department of Health). The Municipality of Svendborg also plans to disseminate its knowledge to interested parties, including other municipalities. It has previously, with great success, conducted conferences featuring the results from the sports school project (The CHAMPS Study-DK) with attendance from many Danish municipalities (more than 300 participants).

## Discussion

The trial within this research program can have far-reaching perspectives because:

- *End user involvement:* The extensive involvement of the end users in the development of the intervention, combined with the Municipality’s commitment to the project, renders its implementation a high chance of success. It is the explicit intention of the Municipality and preschools in Svendborg to implement interventions shown to have a positive impact on the lives of children.
- *The field experiment design:* The design where the intervention is applied at several institutions, all with different physical conditions and cultures, combined with a thorough process evaluation, makes the results transferrable to other municipalities in Denmark and therefore, the potential for impact in practice is considerable.
- *The broad target population:* Interventions in preschools target children and families from all levels of society and therefore have large potential for improving the lives of socially disadvantaged children, who are otherwise difficult to reach with health promoting campaigns and initiatives.

Equally important, the cohort which will be initiated within this program will open up possibilities for future research into the influence of early life factors on future health, lifestyle, academic performance, language development, and much more. Some of these are described in the work packages in this application, but extensive additional use of data both nationally and internationally is anticipated.

Finally, this project distinguishes itself by its broadness of scope, opening up possibilities to investigate interactions and mediating processes across the different domains.

## Trial status

Recruitment started in August 2016 and will continue throughout 2017 for the cohort. However, inclusion into the RCT will be terminated by the January 31, 2017, as this represents the completion of baseline data collection.

By December 19, 2016, the parents of 865 children (59%) had agreed to participate in the study, and 485 of these attended preschools included in the RCT (67% of the eligible RCT children).

## Additional files

Additional file 1: Work packages 1–8. (DOCX 236 kb)

Additional file 2: Svendborg Kommune/Municipality of Svendborg. (DOCX 71 kb)

Additional file 3: SPIRIT 2013 Checklist: Recommended items to address in a clinical trial protocol and related documents*. (DOC 121 kb)

## Abbreviations

BMI: Body mass index
DK: Denmark
DSSCB: Department of Sports Science and Clinical Biomechanics
MABC-2: Movement Assessment Battery for Children, version 2
MiPS: Motor Skills in PreSchool
OPEN: Odense Patient data Explorative Network
RCT: Randomized controlled trial
SDU: SydDansk Universitet (University of Southern Denmark)
UCL: University College Lillebaelt
WP: Work package
YMCA: Young Men’s Christian Association
