# Supplementary material for: Influence of motor skills training on children’s development evaluated in the Motor skills in PreSchool (MiPS) study-DK: study protocol for a randomized controlled trial, nested in a cohort study
Source: Trials. 2017 Aug 29;18:400. doi: 10.1186/s13063-017-2143-9 (PMC5576290; doi:10.1186/s13063-017-2143-9)
Supplement: Supplementary file 1 — Work packages 1–8. (DOCX 236 kb) [file 13063_2017_2143_MOESM1_ESM.docx]

**Additional file 1**

**Work Packages 1-8**

# WP 1: Effectiveness of a structured intervention for improving motor skills in Danish preschool children. A randomised controlled trial.

## Background

There are reports of randomised controlled trials indicating that children’s motor skills can be improved through interventions in preschool, but the study populations have been small, the intervention periods short (<12 weeks), and the follow-up periods less than half a year(1). Thus, there is an urgent need for high quality studies involving larger samples and longer intervention and follow-up periods (see main body of the protocol).

Besides the importance of a child´s actual fundamental movement skills, perceived motor competence seems to be important in supporting physically active behaviour, and it is thought to be a key motivational factor in supporting engagement in physical activities and sports(2-4). Perceived motor competence is defined as children´s self-perceptions about their capabilities in physical or motor domains (5, 6). Although children have unrealistic perceptions of actual competence during their early years, Stodden and colleagues argue that a perceived high level of motor skills might be one of the reasons to engage in physical activities during the early childhood years. The hypothesis is that children with low perceptions of competence find physical activity less enjoyable than their peers with higher perceptions of competence and thus are less likely to be physically active and may be drawn into a negative spiral of disengagement (2).

Furthermore, children with high perceptions of competence are likely to select challenging tasks, enjoy the learning process, exhibit higher self-esteem, exert greater effort to master skills, and persist in the face of difficulty (3, 7).

### Objectives

The primary objective of this work package is to determine the effectiveness of an adaptable but structured intervention to improve motor skills and perceived motor competence in preschool children.

### Intervention

See main protocol and Appendix 2.

### Variables

*Primary outcome:*

Motor skills as assessed by the MovementABC-2 (as described in the main protocol) at the 18 month follow-up (spring 2018). At this point, the children will have received the intervention for 12 months after a six months phase-in period.

*Secondary outcomes:*

Child’s perceived physical competence

The perceived motor competence relating to everyday activities will be measured by the Perceived Physical Competence (PPC) subscale of Harter and Pike’s Pictorial Scale of Perceived Competence and Social Acceptance (PSPCSA) for preschool- and kindergarten-age children (4 to 7 years)(5). The PPC subscale assesses the children’s self-perceptions related to their ability to run, hop, swing, climb, tie shoelaces, and skip. On each pictorial plate, two pictures will be displayed side by side; one picture depicts a child who is competent in a particular task, and the other depicts a child who is not competent. The child selects the picture that is most like him/herself. Then, the child focuses on the selected picture and indicates whether

(s)he is ‘‘a little bit like’’ the child in the picture or ‘‘a lot like’’ the child in the picture. The range of scores for each item on the subscale is 1 (low competence) to 4 (high competence). The instrument has an internal consistency for the individual PPC subscale for preschool children of 0.62 (5). The assessment of the PPC subscale takes around 5 minutes.

Parent-perceived motor competence

The rating of parents’ perception of their child’s athletic coordination compared to other children of the same age has often been related to preschool-age children´s actual physical activity (9-11). An often used question for this assessment is “Compared with other children of the same age and sex, how would you describe your child’s level of athletic coordination?” with a response on a 5-point Likert scale, from 1 (much less coordinated) to 5 (much more coordinated) (10, 11). This question will be included in the parental baseline questionnaire and also mailed to the parents at the time of the last follow up before the child leaves preschool.

## Length of follow up

The duration of the follow up is 6 to 30 months, which covers the period from the beginning of the intervention until the last measurement before the child leaves preschool (see Figure 1 in the main protocol).

## Analyses of effect

Simple univariate statistics will be used to describe the outcomes and covariates by preschool. Bivariate analysis adjusted for the design effect will be done to determine if there were any imbalances in the baseline covariates among the two groups.

Multilevel linear regression models will be constructed to assess the effectiveness of the intervention on motor skill performance. The baseline value of the outcome variable, time since baseline, intervention group and any imbalanced covariates will be added to the model along with preschool and socioeconomic status of the area as random effects. The interaction between preschool and intervention group will be tested. The assumptions for the models will be checked.

Multilevel ordinal regression models will be constructed to assess the effectiveness of the intervention on the parents’ perception of their child’s motor competence. The baseline value of the parents’ perception, time since baseline, intervention group and imbalanced covariates will be independent variables. The school and socioeconomic status of the area will be entered as random effects and the interaction between school and intervention will be tested. The model’s assumptions will be tested.

The above analyses will be repeated on a subsample of children who were considered to have a ‘poor’ level of motor competence at baseline.

An analysis will be conducted to determine if the children who dropped out of the study were different from those who remained, based on the baseline values of the outcome variables and the covariates.

## Perspectives

The Svendborg City Council has granted generous resources to educate the staff; the staff at the preschools is eager to participate; the preschool teachers have contributed to the design of the intervention, ensuring a sense of ownership and involvement; and there is experience with conducting large, cluster randomised controlled trials in the Municipality. Therefore, the conditions for implementation of the intervention in the preschools are optimal. Consequently, if we cannot show an improvement in the children’s motor skills using this framework, further allocation of resources to programs to improve motor development in preschools should probably be discontinued.

On the other hand, if the intervention does succeed, the program can easily be applied in other Danish municipalities due to the design, where each preschool applies the intervention principles in a way to suit their individual conditions regarding physical environment, staffing, etc.

Finally, if the intervention improves motor competence in the children with the highest need for improvement, targeted interventions could be considered if, in some settings, prioritizing of resources is necessary.

## Primary responsibility

Lise Hestbæk, Associate Professor, Department of Sports Science and Clinical Biomechanics, University of Southern Denmark. Senior researcher, Nordic Institute of Chiropractic and Clinical Biomechanics.

## References

1. Riethmuller AM, Jones R, Okely AD. Efficacy of interventions to improve motor development in young children: a systematic review. Pediatrics. 2009;124(4):e782-92.
2. Stodden DF GJ, Langendorfer SJ, Roberton MA, Rudisill ME, Garcia C, Garcia LE. A developmental perspective onthe role of motor skill competence in physical activity: An emergent relationship. Quest. 2008;60:290-306.
3. weiss MR AA. Children's self-perceptions in the physical domain: Between- and within-age variability in level auracy and sources of perceived ompetence. Journal of Sport and Exerise Psychology. 2005;27:226-44.
4. Barnett LM, Morgan PJ, van Beurden E, Beard JR. Perceived sports competence mediates the relationship between childhood motor skill proficiency and adolescent physical activity and fitness: a longitudinal assessment. The international journal of behavioral nutrition and physical activity. 2008;5:40.
5. Harter S, Pike R. The pictorial scale of perceived competence and social acceptance for young children. Child Dev. 1984;55(6):1969-82.
6. Horn T. Developmental perspectives on self-perceptions in children and adolescents. MR W, editor. Morgantown, WV: Fitness Information Technology; 2004.
7. Harter S. Effetance motivation reconsidered. Human Development. 1978;21:34-64.
8. Barnett LM, Ridgers ND, Zask A, Salmon J. Face validity and reliability of a pictorial instrument for assessing fundamental movement skill perceived competence in young children. Journal of science and medicine in sport / Sports Medicine Australia. 2015;18(1):98-102.
9. Loprinzi PD, Trost SG. Parental influences on physical activity behavior in preschool children. Prev Med. 2010;50(3):129-33.
10. Dowda M, Pfeiffer KA, Brown WH, Mitchell JA, Byun W, Pate RR. Parental and environmental correlates of physical activity of children attending preschool. Arch Pediatr Adolesc Med. 2011;165(10):939- 44.
11. Pfeiffer KA, Dowda M, McIver KL, Pate RR. Factors related to objectively measured physical activity in preschool children. Pediatric exercise science. 2009;21(2):196-208.

# WP 2: Process Evaluation

## Background

The rationale for including a process evaluation is that it creates the possibility to gain insight into what happens in local implementation processes. Even the most efficient efforts will, in practice, not yield signifi- cant impacts if they are not implemented with sufficient quality. The intervention to improve children´s motor skills is described in general in Appendix 2. It is up to local stakeholders to interpret the content in order to further operationalize various key components and thus be able to implement the intervention (1).

Overall, the process evaluation builds on the work of Michael Q. Patton, which deals with so-called utilization-focused evaluation. This approach enables the monitoring of activities and outputs to deliver continuous and solid feedback to those directly involved in the intervention and other key stakeholders.1 In later stages, the process evaluation will contribute to generating information about intervention dose and quality, e.g. dose-delivered, dose-received, consistency, appeal and fidelity.

The process evaluation will be guided by the RE-AIM framework (2). This ensures a holistic assessment of intervention impacts - focusing not solely on effectiveness, but also on the process of delivery and how, and to what degree, the main intervention components are institutionalized. For more than fifteen years, RE-AIM has been widely used to enhance the quality, speed, and public health impact of efforts to translate research into practice.

## Objective

To assess the implementation aspects of the intervention to improve motor skills.

## Variables

The RE-AIM framework consists of five fundamental questions:

**Reach:** How many in the overall target group can and are actually willing to participate in the intervention?

**Efficacy:** What kind of main and broader effects are to be expected? Information on the positive and negative effects (intentional or not) of an intervention is included.

**Adoption – setting level:** How many potential settings are willing and/or able to participate?

**Adoption – staff level:** Can and will those who are supposed to execute the intervention actually commit themselves? The capacity and motivation of different professional groups to engage in a given intervention, and specific work modes and tools developed for this, are regarded as crucial for the actual reach and impact of settings-based interventions. Based on the available evidence it must, as far as possible, be explored which barriers and facilitators are in play to move the professional use of effective interventions further.

1 Stakeholders are individual or collective actors having an interest in a given project and/or potentially being affected by its deliverables. In this research program focus is on internal actors interested in and/or affected by the described intervention. External stakeholders encompass all actors having a stake in the program and its delivery, without taking active part in any intervention components and activities (3).

**Implementation:** How consistently is the intervention, and its various parts, delivered? Fidelity issues of the intervention come into focus; if the intervention went as planned; what actually happened, and why; and if the effects corresponded to what was expected and intended. A further important point is to what degree the intervention is modified over time.

**Maintenance – individual and setting level:** Can a given intervention and its effects be maintained over time? This point is often decisive in connection with an overall evaluation of an intervention. In relation to the individual end user, the maintenance issue is about the ability to keep up changes in health-related habits and routines promoted by a given intervention. At the organizational level, the maintenance issue centers on, for instance, the feasibility of making the intervention part of day-to-day operations.

The table below illustrates how the RE-AIM framework will be further primed in this particular project.

| **Element Level Description Measure Assessment** | | | | | |
| --- | --- | --- | --- | --- | --- |
| **Reach**  Individual  (target population)  The impact of an  intervention on pri- mary and secondary  outcomes  CF. Information on  *Variables* and *Anal- yses of effect* for WP 1  CF. Information on  *Variables* and *Anal- yses of effect* for WP 1  Setting &  Individual (target staff)  The number and rep- The target staff and Direct observations  resentativeness of institutions are com- and e-surveys. Barri- preschools that adopt pared to the larger ers and facilitators to the intervention ‘denominator’ popu- adoption will also be  lation. assessed.  Number intervention  staff that adopt the | Individual The number and characteristics of  (target population) participants that re-  ceive, or are affected by the intervention | | | The target population Demographic infor-  is compared to the mation on nonpartici- larger ‘denominator’ pants as well as par- population. ticipants. | |
| **Efficacy**  **Adoption** |  | | | | |
|  | Program | | | | |
| **Implementation**  **Maintenance** | Setting & Individual  (target population) (target staff) | Protocol fidelity, the consistency and skill of how the various intervention compo- nents are delivered by target staff.  Satisfaction with and understanding of significant interven- tion outcomes as- sessed by the target population, together with their next of kin. | Barriers and facilita- tors reported by tar- get staff and target population and their next of kin during implementation | | Field notes and inter- views examining im- plementation issues, with target staff, reached children and their next of kin. |
|  | Setting & Individual (target staff) | Long-term effects on outcomes measures at the individual level  The extent to which the intervention be- comes institutional- ized as part of the daily routines and organizational prac- tices. | Measures the extent to which innovations become an enduring part of the behav- ioral repertoire of the preschool setting. | | Direct observations, structured interviews and e-surveys |

## Analyses of effect

Preschool staff, preschool managers and relevant parts of the municipal administration and management in Svendborg, will be identified and included in the process evaluation. Key methods will be participant observation of practice, individual and group interviews and e-surveys. The children’s perspective will be explored via observation studies and protocol-based assessments of the preschool’s social and pedagogical environments. Participant observation and go-along interviews are applied to capture the children’s experiences (4-6).

The data collection will begin in the spring of 2016 and continue simultaneously with the local implementation processes until the spring of 2019.

Perspectives

RE-AIM makes up a key component in the compiled framework on Knowledge Translation (KT) – which in health sciences is generally defined as a dynamic process that includes synthesis, exchange and application of knowledge to improve health and wellbeing and provide more effective services (7-8). The KT approach is further informed by Knowledge-to- Action (K2A) approaches developed over a number of years by the Canadian Institutes of Health Research (CIHR) (9). Together K2A and RE-AIM form a systematic and well developed methodological toolbox to support integration of best available research evidence with local context and practice and, finally, to feed back to the general knowledge base. For this particular project, it is of value to differentiate between two types of knowledge translation processes – namely *integrated and final KT.* The latter is concerned with promoting the findings, experiences and results of the research project. Key messages for communication and dissemination are developed to facilitate transfer of knowledge, targeting specific audiences. Integrated KT, also found under the headings of co-creation of knowledge and collaborative research, refers to the processes in which KT is installed as a continued activity to ensure that all project phases are based on the best available empirical and theoretical knowledge by facilitating close interaction between researchers, end users and other relevant stakeholders (9).

The basic premise is that KT should be utility-driven in the sense that knowledge generated from research (constituting one type of information) is only complete when it informs stakeholders on matters of relevance and importance, thereby assisting in the development, modification and planning of current and future interventions.

## Primary responsibility

Professor Jens Troelsen, Research Unit for Active Living, Department of Sports Science and Clinical Biomechanics, SDU

Associate Professor Thomas Skovgaard, Research Unit for Active Living , Department of Sports Science and Clinical Biomechanics, SDU.

## References

1. Krogstrup, H.K. (2006). Evalueringsmodeller. Aarhus: Academica.
2. Glasgow, R. E., Vogt, T. M., & Boles, S. M. (1999). Evaluating the public health impact of health promotion interventions: The RE-AIM framework. American Journal of Public Health, 89(9), 1322-1327.
3. Friedman, A.L., Miles S. (2006) Stakeholders: Theory and Practice: Theory and Practice, Oxford University Press
4. Højlund, S. (2002). Barndomskonstruktioner. På feltarbejde i skole, SFO og på Sygehus. København: Gyldendal
5. Gulløv, E., Højlund, S. (2006). Feltarbejde blandt børn. Metodologi og etik i etnografisk børneforskning. København: Gyldendal
6. Ballegaard, S. A., Grøn, L., & Olesen, E. (2013). Del 3: Et brugerperspektiv på Space. In J. Troelsen (Ed.), SPACE - Rum til fysisk aktivitet. Organisatorisk evaluering og brugerperspektiv på en helhedsorienteret, forebyggende indsats for børn og unge. Delrapport, januar 2013 (pp. 96-133). Odense: Syddansk Universitet
7. Satterfield, J. M., Spring, B., Brownson, R. C., Mullen, E. J., Newhouse, R. P., Walker, B. B., & Whitlock, E. P. (2009). Toward a Transdisciplinary Model of Evidence-Based Practice. *The Milbank Quarterly*, *87*(2), 368– 390.
8. Kessler R., Glasgow R. E. (2011) A proposal to speed translation of healthcare research into practice: dramatic change is needed. Am J Prev Med. 2011 Jun;40(6):637-44
9. Straus, S. E., Tetroe, J., & Graham, I. (2009). Defining knowledge translation. *CMAJ : Canadian Medical As- sociation Journal*, *181*(3-4), 165–168.

# WP 3: Motor performance and musculoskeletal disorders

### Background

Intuitively, motor skills and musculoskeletal (MSK) disorders are linked, but the reality is that a knowledge gap exists and the association between the two is unclear at present. MSK disorders are now the largest contributor to the global burden of disease (1) and the leading somatic cause of disability in Denmark (2). Research published over the last decade has consistently shown that MSK disorders start early in life (3-5), and that back pain in adolescence leads to back pain in adulthood (4). However, why and how early MSK disorders and back pain originates is unknown because, so far, studies have mainly focused on children aged 12 years or older. Importantly, the immediate consequences of poor MSK health early in life appear to be extensive. For example, children experiencing pain may miss school and withdraw from sport and other social activities and are at risk of the developing negative health behaviours such as physical inactivity, when compared to children with good MSK health. This may in turn increase the risk of lifestyle disorders later in life such as cardiovascular disease or diabetes (6, 7). In addition, appropriate use of the MSK system is likely to decrease the risk of both overuse injuries (8, 9), which appear to be relatively prevalent in younger children (10). Also, the risk of traumatic injuries might be reduced (11) and several motor control interventions have been shown to decrease the risk of traumatic injuries among adolescents and military recruits (12-14).

Surprisingly, the importance of regular motor skills training of preschool children in terms of developing MSK problems has never been investigated before. However, there is good reason to believe that improved motor performance may have a positive effect on preventing problems of MSK origin. Firstly, several studies of focused motor performance training have shown an increase in muscle control, coordination and balance in adults (15-17). In addition, several interventions designed to improve motor development in young children have been shown to be effective (18) even at three years’ follow-up (19). Secondly, motor performance training of adults has revealed improved activation of the abdominal musculature especially when challenged by sudden trunk perturbations (20, 21). This is relevant, as children with poorly developed motor abilities (Developmental Coordination Disorder) often show poor activity in these muscle groups (22). Thirdly, it is likely that motor performance training has a positive effect on the development of ‘normal’ movement patterns, resulting in appropriate functioning of mechanical joints and muscle load.

Finally, studies indicate that improved motor control can reduce the frequency of MSK injuries in the extremities (12-14).

High quality studies investigating the potential benefit of improved motor skills on MSK health are urgently needed. A prophylactic strategy focusing on improving motor skills in preschool children may potentially reduce the prevalence of current and future risk of MSK injuries and the consequences thereof.

### Aim

The long-term aim is to improve the life course of MSK disorders through identification of target areas for early prevention.

### Objectives to be explored in the RCT

- 1. To estimate the effect of a motor skills intervention on aberrant movement patterns and musculoskeletal health.

### Objectives to be explored in the full cohort

1. To describe the age and sex related incidence and course of back-, neck- and extremity-disorders in a general paediatric population from three to fifteen years of age.
2. To establish potential patterns of development of musculoskeletal disorders from three to fifteen years of age.
3. To determine the potential relationship between poor motor performance and aberrant movement patterns.
4. To determine the potential relationship between motor performance at the end of preschool and musculoskeletal health throughout the school years.

### Variables

Predictors

- Motor performance as described under ‘core outcomes for all WP’s’
- Movement patterns will be evaluated by kinematic analyses of Drop Vertical Jump Test and Standing Broad Jump Test.

*Drop Vertical Jump Test*

The Drop Vertical Jump Test (DVJ) is a validated clinical test, in which kinematics around the knee are assessed during an accelerated vertical jump from a 31-cm. high box, landing on both feet and immediately followed by vertical jump as high as possible (11). Aberrant movements, including increased valgus motion, high abduction loads, and increased internal rotation of the hip, has repeatedly been shown as a risk factor for (a) anterior cruciate ligament rupture among female athletes (11), and (b) development of patella femoral pain syndrome among female athletes (23, 24) and military recruits of both sexes (25).

*Standing Broad Jump Test (a.k.a. Horizontal Long Jump Test)*

The Standing Broad Jump Test (SBJ) is performed by having the child stand behind a line on the floor, jumping as far as possible, and the length of the jump is measured. It is considered a simple, cheap and safe test, suitable for the assessment of lower body maximal strength in adolescents (26). SBJ has been tested on children as young as 5 years of age, and found to have normally distributed total scores and good reliability (ICC=0.88) (27). Furthermore, it has been used to monitor children with either low or high motor competence with significant discriminative abilities over a 5 years’ period (28).

*Measurement equipment*

The DVJ and SBJ tests will be measured using the Captury motion capture system ([www.thecaptury.com](http://www.thecaptury.com)). The Captury is a portable 8-camera high-speed motion capture system which makes no use of any markers, suits or special hardware. A child avatar has been specially developed to be used in the present project in collaboration with Institute of Sports Science and Clinical Biomechanics, University of Southern Denmark. The Captury has been found to be reliable compared to the Vicon motion capture system used as gold standard (unpublished data, article under preparation).

Outcome

The presence of complaints from the MSK system will be reported by SMS-track. Parents will receive text messages (SMS) on a biweekly basis inquiring about the child´s MSK pain as well as observations of abnormal behaviour the past week. Replies are automatically registered and stored in an encrypted database. The system has been in use in another project in the schools of Svendborg Municipality where more than 95% of the participating families responded each week [32]. A validation study was undertaken in order to determine the reproducibility of the SMS-track reporting when comparing it with verbal reporting. The sensitivity for the SMS data was 0.98, specificity 0.87, positive predictive value 0.94 and negative predictive value 0.95, indicating high validity of data [33].

The SMS questions were developed on the basis of a qualitative study investigating how parents to children aged 3-6 years become aware of their children’s MSK pain. The study derived six themes: ‘wont/can’t use part of the body’, ‘physical signs’, ‘communicate it’, ‘demonstrate it with an outburst’, ‘change of behaviour’, and ‘protect the painful area’. A nominal group technique was used to generate the initial SMS questions and these were subsequently tested in a pilot study. The final SMS questions were:

***Introductory SMS:***

“Dear Parents.

Thank you for participating in the project Active Children in Daycare.

In the future, you will receive 1 or 2 SMS’s every second Sunday with questions about [*child name*] muscle and joint pain in the previous 2 weeks. Pain in muscles and joints in children can be detected in many ways. Your child may tell or show it to you, hold the area or protect it and not let anyone touch it. Maybe he/she will use the body in a different way (e.g. avoiding normal movements, limp or not use one arm). It is important that you answer all SMS’s, even if [*child name*] has not had any pain.

If you have any questions to the SMS’s, please contact project coordinator [*name*] on [*telephone number*].”

***SMS 1 (every second Sunday):***

Do you think you child have had pain from muscles or joints in the past two weeks?

Answer 1 for yes and 2 for no.

***SMS 2 (only send out if answering ‘yes’ to SMS 1):***

How did you detect it? (Answer with numbers. You can use multiple answer options by using a comma between the numbers, e.g. 1,2)

1. [*Child name*] told or showed me

2. [*Child name*] changed the use of his/her body/arm/leg or other body part

3. [*Child name*] have had a changed mood/behaviour/sleep

Both questions will be asked during the preschool years. If the same problem is reported two consecutive times, a research assistant will call the parents and offer examination and treatment by an orthopaedic surgeon at the sports clinic at the Odense University Hospital.

Thus, the three outcomes used for analyses will be:

- - Parent-reported pain
  - Parent-reported detection of problems
  - MSK diagnoses of persistent problems

### Covariates

The following covariates will be accounted for when analysing the results: sex, age, socioeconomic status, child well-being and parental MSK health.

### Length of follow-up

The duration of the follow-up will be 12 years, which covers three years in preschool and nine years in school.

### Analyses

Multilevel logistic regression will be conducted to determine if there was a difference in aberrant movement problems by intervention group after adjusting for time in intervention, imbalanced covariates from WP1, school and socioeconomic status of the school.

Multilevel Poisson regression will be conducted if the number of MSK diagnoses of persistent problems differs by intervention group after adjusting for time on intervention, imbalanced covariates from WP1, school and socioeconomic status of the school.

The age-specific, sex-specific and age- and sex-specific incidence rate of MSK conditions will be calculated.

Longitudinal latent class analysis and latent class growth analysis will be conducted to describe the MSK developmental patterns. The sequential model comparisons (T classes vs. T + 1 classes) and chi-square goodness-of-fit test will be evaluated using the parametric bootstrapping technique. Lastly, the associations between class membership and the prevalence of related measures will be examined using a confirmatory latent class analysis approach.

Multilevel longitudinal logistic regression analyses will be conducted to determine if poor motor performance is associated with aberrant movement patterns after adjusting for age, sex and other important covariates. The interaction between time and poor motor performance will be examined.

Multilevel longitudinal Poisson regression analyses will be conducted to determine if motor performance at the end of preschool is predictive of the number of MSK diagnoses throughout the school years after adjusting for age, sex and other important covariates. The interaction between time and motor performance will be examined.

**Perspectives**

The RCT has the potential to show the effect of a motor performance programme on specific movement patterns. Of equal importance, the project will bring new insights into the early phases of MSK problems, including how and why these problems arise and develop and the type of problems children from three to 15 years of age experience. Through this rich dataset, we will be able to identify and recognise risk patterns which are important for preventing future chronic MSK conditions. Together, this opens ‘a window of opportunity’ for implementing targeted prophylactic strategies with the potential to reduce the risk of current and future MSK injuries and their associated individual and societal burden.

## Primary responsibility

Henrik Hein Lauridsen, Associate Professor, Dept. of Clinical Biomechanics, University of Southern Denmark

### References

1. Vos T, Flaxman AD, Naghavi M, Lozano R, Michaud C, Ezzati M, et al. Years lived with disability (YLDs) for 1160 sequelae of 289 diseases and injuries 1990-2010: a systematic analysis for the Global Burden of Disease Study 2010. Lancet. 2012;380(9859):2163-96.
2. Institute for Health Metrics and Evaluation (IHME). GBD Cause Patterns. Seattle WI, 2013. UoW. Available from: [http://www.healthdata.org/data-visualization/gbd-cause-patterns.](http://www.healthdata.org/data-visualization/gbd-cause-patterns)
3. Jeffries LJ, Milanese SF, Grimmer-Somers KA. Epidemiology of adolescent spinal pain: a systematic overview of the research literature. Spine. 2007;32(23):2630-7.
4. Hestbaek L, Leboeuf-Yde C, Kyvik KO, Manniche C. The course of low back pain from adolescence to adulthood: eight-year follow-up of 9600 twins. Spine. 2006;31(4):468-72.
5. Aartun E, Hartvigsen J, Wedderkopp N, Hestbaek L. Spinal pain in adolescents: prevalence, incidence, and course: a school-based two-year prospective cohort study in 1,300 Danes aged 11-13. BMC musculoskeletal disorders. 2014;15:187.
6. Andersen LB, Bugge A, Dencker M, Eiberg S, El-Naaman B. The association between physical activity, physical fitness and development of metabolic disorders. International journal of pediatric obesity : IJPO : an official journal of the International Association for the Study of Obesity. 2011;6 Suppl 1:29-34.
7. Froberg K, Andersen LB. Mini review: physical activity and fitness and its relations to cardiovascular disease risk factors in children. International journal of obesity. 2005;29 Suppl 2:S34-9.
8. Wilder RP, Sethi S. Overuse injuries: tendinopathies, stress fractures, compartment syndrome, and shin splints. Clin Sports Med. 2004;23(1):55-81, vi.
9. Niemuth PE, Johnson RJ, Myers MJ, Thieman TJ. Hip muscle weakness and overuse injuries in recreational runners. Clin J Sport Med. 2005;15(1):14-21.
10. Jespersen E, Holst R, Franz C, Rexen CT, Klakk H, Wedderkopp N. Overuse and traumatic extremity injuries in schoolchildren surveyed with weekly text messages over 2.5 years. Scandinavian journal of medicine & science in sports. 2014;24(5):807-13.
11. Hewett TE, Myer GD, Ford KR, Heidt RS, Jr., Colosimo AJ, McLean SG, et al. Biomechanical measures of neuromuscular control and valgus loading of the knee predict anterior cruciate ligament injury risk in female athletes: a prospective study. Am J Sports Med. 2005;33(4):492-501.
12. Pasanen K, Parkkari J, Pasanen M, Hiilloskorpi H, Makinen T, Jarvinen M, et al. Neuromuscular training and the risk of leg injuries in female floorball players: cluster randomised controlled study. Bmj. 2008;337:a295.
13. Parkkari J, Taanila H, Suni J, Mattila VM, Ohrankammen O, Vuorinen P, et al. Neuromuscular training with injury prevention counselling to decrease the risk of acute musculoskeletal injury in young men during military service: a population-based, randomised study. BMC medicine. 2011;9:35.
14. Schiftan GS, Ross LA, Hahne AJ. The effectiveness of proprioceptive training in preventing ankle sprains in sporting populations: A systematic review and meta-analysis. Journal of science and medicine in sport / Sports Medicine Australia. 2014.
15. Aman JE, Elangovan N, Yeh IL, Konczak J. The effectiveness of proprioceptive training for improving motor function: a systematic review. Frontiers in human neuroscience. 2014;8:1075.
16. Smith TO, King JJ, Hing CB. The effectiveness of proprioceptive-based exercise for osteoarthritis of the knee: a systematic review and meta-analysis. Rheumatology international. 2012;32(11):3339-51.
17. Aasa B, Berglund L, Michaelson P, Aasa U. Individualized low-load motor control exercises and education versus a high-load lifting exercise and education to improve activity, pain intensity, and physical performance in patients with low back pain: a randomized controlled trial. The Journal of orthopaedic and sports physical therapy. 2015;45(2):77-85.
18. Riethmuller AM, Jones R, Okely AD. Efficacy of interventions to improve motor development in young children: a systematic review. Pediatrics. 2009;124(4):e782-92.
19. Zask A, Barnett LM, Rose L, Brooks LO, Molyneux M, Hughes D, et al. Three year follow-up of an early childhood intervention: is movement skill sustained? Int J Behav Nutr Phys Act. 2012;9:127.
20. Vera-Garcia FJ, Elvira JL, Brown SH, McGill SM. Effects of abdominal stabilization maneuvers on the control of spine motion and stability against sudden trunk perturbations. Journal of electromyography and kinesiology : official journal of the International Society of Electrophysiological Kinesiology. 2007;17(5):556-67.
21. Grenier SG, McGill SM. Quantification of lumbar stability by using 2 different abdominal activation strategies. Archives of physical medicine and rehabilitation. 2007;88(1):54-62.
22. Kane K, Barden J. Frequency of anticipatory trunk muscle onsets in children with and without developmental coordination disorder. Physical & occupational therapy in pediatrics. 2014;34(1):75-89.
23. Myer GD, Ford KR, Foss KD, Rauh MJ, Paterno MV, Hewett TE. A predictive model to estimate knee-abduction moment: implications for development of a clinically applicable patellofemoral pain screening tool in female athletes. J Athl Train. 2014;49(3):389-98.
24. Myer GD, Ford KR, Barber Foss KD, Goodman A, Ceasar A, Rauh MJ, et al. The incidence and potential pathomechanics of patellofemoral pain in female athletes. Clinical biomechanics. 2010;25(7):700- 7.
25. Boling MC, Padua DA, Marshall SW, Guskiewicz K, Pyne S, Beutler A. A prospective investigation of biomechanical risk factors for patellofemoral pain syndrome: the Joint Undertaking to Monitor and Prevent ACL Injury (JUMP-ACL) cohort. Am J Sports Med. 2009;37(11):2108-16.
26. Bianco A, Jemni M, Thomas E, Patti A, Paoli A, Ramos Roque J, et al. A systematic review to determine reliability and usefulness of the field-based test batteries for the assessment of physical fitness in adolescents - The ASSO Project. Int J Occup Med Environ Health. 2015;28(3):445-78.
27. Fjortoft I, Pedersen AV, Sigmundsson H, Vereijken B. Measuring physical fitness in children who are 5 to 12 years old with a test battery that is functional and easy to administer. Phys Ther. 2011;91(7):1087-95.
28. Hands B. Changes in motor skill and fitness measures among children with high and low motor competence: a five-year longitudinal study. Journal of science and medicine in sport / Sports Medicine Australia. 2008;11(2):155-62.
29. **WP 4: Motor skills influence on physical activity and overweight, and population-based motor skills**
30. **reference data**
31. **Background**
32. Engaging repeatedly in various physical activities in early childhood is fundamental for motor skill
33. development. However, poorly developed motor skills may also cause a propensity for choosing habitual
34. activities that are sedentary in nature and a lack of engagement in sports participation in the short and long
35. term, resulting in a vicious cycle. Physical inactivity is a principal risk factor for many poor health conditions,
36. chronic non-communicable diseases, and may ultimately influence longevity. Hence, promotion of physical
37. activity in early life is a critical public health issue. Because children do not necessarily grow out of their
38. motor skill difficulties in early childhood, it is important to closely examine the relationship between motor
39. skills and physical activity from this early stage of life. Understanding the relationship between the two
40. constitutes an essential element needed to inform health and educational authorities as to the reasons for
41. prioritizing motor skill-oriented activity in early childhood.
42. To date, the potential nature of the relationship between motor skills and physical activity in preschool-age
43. children has received little scientific attention. When reviewing the literature, it becomes readily apparent
44. that the majority of the results from past studies are based on cross-sectional data. Furthermore,
45. inconsistent and limited findings make it difficult to determine whether motor skills during early childhood
46. predict habitual physical activity or sports participation later in childhood (1-3). In fact, we are only aware
47. of one study examining the relationship between motor skills during the preschool years and physical
48. activity in later childhood that has used an objective measure of physical activity. This study found that
49. motor skills were unrelated to later physical activity; however, the study was very modest in size and had a
50. short follow-up period (9 months) (1). Randomized controlled trials carried out in the preschool setting
51. with the aim of improving motor skills in healthy preschool children have not examined short- or long-term
52. effects on physical activity (4-6). Thus, the evidence base currently available to understand the extent to
53. which poor motor skills acquisition in early childhood causes later physical inactivity is limited.
54. Another important and plausible consequence of poorly developed motor skills in early childhood is
55. obesity. Although this relationship to a large extent could be explained by habitually low physical activity, it
56. is important to establish the direct and indirect effects of motor skill development on obesity in early
57. childhood. The scientific evidence for this topic is also very limited, and randomized controlled trials and
58. appropriately sized cohort studies are warranted - this being another important reason for conducting this
59. project. Finally, because the sample in the current study is community-wide, it is a valuable source for
60. obtaining reference data on motor skills according to the MABC-2 motor skills test battery. Such reference
61. data is valuable for future surveillance and screening purposes. Thus, an additional aim of this WP is to
62. establish a population-based set of motor skill data to provide standards for interpreting values of motor
63. skills by age and sex.
64. **Objectives**
65. Collectively, the objectives of this WP are:
66. 1. To examine the effect of a three-year motor skills intervention program on short-term (<3-year)
67. changes in objectively measured physical activity and body composition in children attending preschool
68. 2. To examine the effect of a three-year motor skill intervention program in preschool children on longterm
69. changes in objectively measured physical activity and sports participation from preschool age to
70. preadolescence
71. 3. To elucidate the possible bi-directional association between motor skills and objectively measured
72. physical activity and sports participation during preschool and in preadolescence
73. 4. To establish population-based reference data on motor skills according to age and sex
74. **Variables**
75. The outcome measures for this WP are objectively measured physical activity and obesity. A description of
76. the methods to assess motor skills and body composition is provided in the main protocol.
77. *Assessment of physical activity*
78. Habitual physical activity will be assessed at baseline, and at 18 and 30 month.
79. Detailed, objective information on the whole spectrum of behaviours (from sleep-cycle to vigorous activity) will be assessed by continuous 24-hour/6 day Axivity® accelerometer measurement using simultaneous thigh and lower back positioned monitors. The method is an age adjusted version of the method used by Skotte et al. (2014) which provides the ability to classify postural allocations like sitting, lying and standing and activities like walking and running with high sensitivity and specificity (7).
80. The Axivity® AX3 monitor is a small (23 x 32.5 x 7.6 mm), lightweight (11 g), waterproof, 3-axis accelerometer data logger (Axivity, 2016). The monitors will be attached on the child´s right thigh and to the right on the lower back using an allogenic adhesive patch to enable full 24-hour recordings.
81. Compared to previously used methods using this method will likely improve compliance rates, improve the control of non-wear time and the quality of measurement since the accelerometer keeps the same position on the body and follows the movement of the body. This method has previously been applied in 900 Danish children 9-14 years of age (8).
82. The instrument will be attached to the body using skin-friendly and permeable adhesive patches. If the children participating in this project have eczema or other skin problems, they will be offered to wear an instrument in a belt.
83. We have previously used accelerometers to assess physical activity among large groups of children in this age-group (9-11). The monitors will be initialized to record data during six consecutive days in raw sampling format, which makes it suitable for detecting the short bursts of activity that are typical of preschool children. Sports participation will be obtained from school age using parental-reported SMS as described in WP 3 and previously obtained in the CHAMPS-DK study (12).
84. *Analysis plan*
85. Mixed model linear regression will be used to compare 3-year changes in physical activity and adiposity
86. between children in the intervention group and control group with appropriate adjustment for imbalanced
87. covariates and baseline levels of the outcomes. Bi-directional association between motor skills
88. development and change in physical activity and sports participation will be analyzed in a cohort approach
89. using mixed model regression with adjustment for experimental allocation group and other putative
90. determinants of motor skills and physical activity.
91. Annually obtained information on motor skills will be used to calculate percentile curves for motor skills
92. from age 3 to 5 years to illustrate the developmental course and provide age- and sex-specific variability in
93. a community-wide sample of apparently healthy children.
94. **Primary responsibility**
95. Associate Professor Anders Grøntved (Head of Research Unit for Exercise Epidemiology and Research in
96. Childhood Health), Department of Sports Science and Clinical Biomechanics, University of Southern
97. Denmark.
98. **References**
99. 1. Burgi F, Meyer U, Granacher U, Schindler C, Marques-Vidal P, Kriemler S, et al. Relationship of physical
100. activity with motor skills, aerobic fitness and body fat in preschool children: a cross-sectional and
101. longitudinal study (Ballabeina). Int J Obes (Lond). 2011;35(7):937-44.
102. 2. Lopes VP, Rodrigues LP, Maia JA, Malina RM. Motor coordination as predictor of physical activity in
103. childhood. Scand J Med Sci Sports. 2011;21(5):663-9.
104. 3. Vandorpe B, Vandendriessche J, Vaeyens R, Pion J, Matthys S, Lefevre J, et al. Relationship between
105. sports participation and the level of motor coordination in childhood: a longitudinal approach. Journal of
106. science and medicine in sport / Sports Medicine Australia. 2012;15(3):220-5.
107. 4. Reilly JJ, Kelly L, Montgomery C, Williamson A, Fisher A, McColl JH, et al. Physical activity to prevent
108. obesity in young children: cluster randomised controlled trial. BMJ. 2006;333(7577):1041.
109. 5. Jones RA, Riethmuller A, Hesketh K, Trezise J, Batterham M, Okely AD. Promoting fundamental
110. movement skill development and physical activity in early childhood settings: a cluster randomized
111. controlled trial. Pediatric exercise science. 2011;23(4):600-15.
112. 6. Krombholz H. The impact of a 20-month physical activity intervention in child care centers on motor
113. performance and weight in overweight and healthy-weight preschool children. Perceptual and motor skills.
114. 2012;115(3):919-32.
115. 7. Skotte J, Korshoj M, Kristiansen J, Hanisch C, Holtermann A. (2014). Detection of Physical Activity
116. Types Using Triaxial Accelerometers. *Journal of Physical Activity & Health*. 11(1):76-84.
117. 8. Schneller, M.B., Bentsen, P., Nielsen, G., Brønd, J.C., Ried-Larsen, M., Mygind, E., & Schipperijn, J. (2016).
118. Measuring Children´s Physical Activity: Compliance Using Skin-taped Accelerometer. Medicine & Science in
119. Sports & Exercise [submitted].
120. 9. Grontved A, Pedersen GS, Andersen LB, Kristensen PL, Moller NC, Froberg K. Personal Characteristics and
121. Demographic Factors Associated With Objectively Measured Physical Activity in Children Attending
122. Preschool. Ped Exerc Sci. 2009;21(2):209-19.
123. 10. Olesen LG, Kristensen PL, Ried-Larsen M, Grontved A, Froberg K. Physical activity and motor skills in
124. children attending 43 preschools: a cross-sectional study. BMC pediatrics. 2014;14:229.
125. 11. Olesen LG, Kristensen PL, Korsholm L, Froberg K. Physical activity in children attending preschools.
126. Pediatrics. 2013;132(5):e1310-8.
127. 12. Hebert JJ, Moller NC, Andersen LB, Wedderkopp N. Organized Sport Participation
128. Is Associated with Higher Levels of Overall Health-Related Physical Activity in Children (CHAMPS Study-DK).
129. PLoS One. 2015;10(8):e0134621.
130. 13. Cole TJ, Green PJ. Smoothing reference centile curves: the LMS method and penalized likelihood. Stat

# Med. 1992;11(10):1305-19.

# WP 5: Associations between early language and motor skills

### Background

The development of early language skills and other cognitive and social skills are correlated in early childhood and have been linked to later academic achievement in numerous studies (for a review see, La Paro & Pianta, 2000). However, the association between motor and language development is not well understood.

In preschoolage and slightly older children, most studies have assessed the role of motor abilities in children with specific language impairment (SLI). Studies have demonstrated that in children with SLI, gross motor skills development is significantly correlated with auditory comprehension, impressive and expressive language skills, and articulation, which collectively indicate a complex relationship between motor and language skills in these children (3, 8, 10). Studies have also demonstrated strong associations between early motor and language development in children with dyslexia (6, 7)

The majority of studies in the general populations have been conducted with infants. A recent systematic review identified 17 studies which demonstrated in different ways significant relationships between the development of motor skills and language and social interactions in typically developing children aged between 1 and 3 years (2). For instance, two large populationbased cohort studies found that in particular fine motor skills and communications skills were significantly correlated and that early motor skills most strongly predicted later communication skills (9) and that infants with minor deviances from normal neuromotor development had a somewhat higher risk of being language delayed (5). There are relatively few studies in typically developing preschoolage children. A study by Cheng et al. (2009) indicated that in 5- 6 year old children motor skills accounted for a significant amount of variance in language measures.

Similarly, a study of 3-6 year old children found that fine motor skills appeared to be the most correlated with language skills, but at 3 and 4 years gross motor skills were also correlated with receptive and expressive language skills (4).

Current findings therefore suggest in particular an association between fine motor skills and language development in typically developing children and that poor or atypical motor development could be an important contributing factor to early language delay. The high correlation and the predictive value of early motor skills on later language development are often interpreted as evidence that motor and language difficulties are two manifestations of a common underlying neurodevelopmental weakness but the relationship between language and motor skills is probably complex and multifaceted (9, 3). However, the findings are based on relatively few studies, in particular in preschoolage children, and the association with long-term reading outcomes has mainly been studied in children with dyslexia. More longitudinal research based on a populationbased sample is therefore needed. Given the associations between language and motor development, a motor performance programme may have impact on language development.

However, existing intervention studies mix language and physical activities (e.g. 1) in a way that it is not possible to measure the specific impact of the motor performance activities on language development.

### Aim

To examine associations between early language and motor skills in typical and atypical children aged 3-5 years and to investigate whether a motor skill intervention can improve language and pre-literacy development in preschool children.

### Objectives to be explored in the RCT

1. To estimate the effect of a motor skills intervention on language and pre-literacy development in preschool children

### Objectives to be explored in the cohort study

1. To determine the cross-sectional association between language and pre-literacy development in preschool children and parent and child characteristics
2. To determine the cross-sectional association between language and pre-literacy development and motor performance in typical and atypical children from age three to six.
3. To establish potential patterns of development of language/pre-literacy development, and motor performance and later reading development from three to fifteen years of age.

### Variables

Outcome:

*Language and pre-literacy development.*

The language and pre-literacy skills of children will be assessed using *Language assessment 3-6* (LA; Bleses et al., 2015) which is administered by the preschool teachers. The assessment battery consists of measures within two domains of language, i.e. oral language (Vocabulary, Comprehension, Communication strategies) and pre-literacy measures (Rhyme detection, Print awareness, Deletion and Letter identification). With the exception of Communication, children will be directly assessed via picture identification or picture elicitation tasks. A validation study shows acceptable data characteristics according to the criteria for the Rasch model for the subscales. Internal consistency coefficients (Cronbach’s alpha) for subscales were between .75-.91; correlations between subscales were between .25-.70. The concurrent correlations of the language subscales with The Peabody Picture Vocabulary Test-4 (PPVT-4, Dunn and Dunn, 2012) were .55 for Vocabulary and .57 for Comprehension; correlations with the Expressive Vocabulary Test 2 (EVT-2, Williams, 2007) were .42 for Vocabulary and .39 for Comprehension. For the pre-literacy subscales, the correlations with PPVT-4 were .33 for Rhyme detection, .49 for Deletion, and .39 for Letter Identification; the correlations with EVT-2 were .18 for rhyme, .29 for Deletion, and .33 Letter Identification.

*Reading measures.* The Danish National Tests includes ten mandatory tests in six subjects, administered individually to children from Grade 2 to Grade 8, offering the opportunity to follow academic achievement of Danish children through the period of compulsory schooling. The reading measure from The Danish National Tests in Grades 2, 4, and 6 will be used. The Danish National Tests in reading evaluate students’ reading ability within three areas: Language comprehension (semantics of individual words, homonyms, language use, idioms), Decoding (word identification in concatenated words, word reading), and Reading comprehension (comprehension of written texts). The Danish National Tests provide a valid estimate of student abilities (Beuchert, & Nandrup 2014). For example, lower scores are associated with low birth

weight, special education needs and low socioeconomic status; all together student and parental background explained approximately 13-21% of the variance in performance. The grade 9 school examinations provided an external measure of validity; when they were regressed on the same-subject national test data, the earlier national test results explained 48-49% of the Danish and math examination marks (Beuchert & Nandrup, 2014).

### Covariates

The following covariates will be accounted for when analysing the results: Sex, age, socioeconomic status, motor development.

### Length of follow-up

The duration of the follow-up will be 12 years which cover three years in preschool and nine years in school.

### Analyses

Cross-sectional analyses will be conducted using multilevel linear regression to determine the association between language and literacy skills and motor development after adjusting for age, gender and SES level.

Multilevel linear regression analysis will be used to determine the prediction from the early language and literacy measures, together with age, gender, and SES, to the three educational outcomes of Language comprehension, Decoding, Reading comprehension, taken from the National Test. The regression analysis determined the effect of age, sex and SES on mean levels of performance, as well as evaluating the unique variance contributed by early language, literacy motor performance and SES.

### Perspectives

The project will reveal new insights into the relationship between early language and pre-literacy development and motor development in both typical and atypical populations, and the development of language/pre-literacy development, and motor performance and later reading development from three to fifteen years of age. The findings may enable recognition of related risk patterns in both language and motor development and affect interpretations of screening test results of early language delay (e.g. in relation to the mandatory language screening of three-year old children in Denmark) and the planning of early (and possible more targeted) interventions for those at risk of motor and language difficulties. The RCT has the potential to show the effect of a motor performance programme on language development in particular in children at risk of language delay. Together, this opens ‘a window of opportunity’ for implementing more global early interventions in daycare centres.

### Primary responsibility

Dorthe Bleses, Professor, Tryg Foundations Centre for Child Research, University of Aarhus

### References

- - 1. Björn, P. M., Kakkuri, I., Karvonen, P., & Leppanen, P. H. T; (2012) Accelerating Early Language Development with Multi-Sensory Training. Early Child Development and Care. 182: 435-451.
    2. Hayley, L. C. & Hill, E. L., (2014) Review: The impact of motor development on typical and atypical social cognition and language: a systematic review. Child & Adolescent Mental Health. 19: 163-170.

*3.* Iverson, J. M.*, &* Braddock, B. A. *(*2006*).* Gesture and motor skill in relation to language in children with language impairment*. Journal of Speech, Language, and Hearing Research,* ***54****, 72–86.*

1. Muluk N., Bayoglu B., Anlar B. (2014) Language development and affecting factors in 3- to 6-year- old children. European Archives of Oto-Rhino-Laryngology. 271: 871-878.
2. van Batenburg-Eddes, T. , Henrichs, J., Schenk, J. J; Sincer, I., de Groot , L. , Hofman, A., Jaddoe, V. W., Verhulst, F. C., Tiemeier, H. (2013). Early infant neuromotor assessment is associated with language and nonverbal cognitive function in toddlers: The Generation R Study. Journal of Developmental and Behavioral Pediatrics. 34: 326-334.
3. van der Leij , Aryan , van Bergen , Elsje , van Zuijen , Titia , de Jong , Peter , Maurits Natasha, Maassen Ben (2013) Precursors of Developmental Dyslexia: An Overview of the Longitudinal Dutch Dyslexia Programme Study. Dyslexia. 19: 191-213.
4. Viholainen, H. Ahonen T, Lyytinen, P., Cantell, M., Tolvanen, A., & Lyytinen, H. (2006) Early motor development and later language and reading skills in children at risk of familial dyslexia. Developmental Medicine & Child Neurology. 48: 367-373.
5. Vukovic, M.*,* Vukovic, I.*, &* Stojanovik, V. *(*2010*).* Investigation of language and motor skills in Serbian speaking children with specific language impairment and in typically developing children*. Research in Developmental Disabilities, 31, 1633–1644.*
6. Wang M V; Lekhal R, Aaro L E; Schjolberg S (2014) Co-occurring development of early childhood communication and motor skills: results from a population-based longitudinal study. Child Care Health and Development. 40: 77-84.
7. Webster, R.I.*,* Majnemer, A.*,* Platt, R.W.*, &* Shevell, M.I. *(*2005*).* Motor function at school age in children with a preschool diagnosis of developmental language impairment*.* The Journal of Pediatrics*,* 146*,* 80*–*85*.*

# WP 6: Association between motor skills and wellbeing

**Background**

Motor skills contribute to children’s physical, cognitive and social development [1]. However, the relationship between motor skills and wellbeing has been less investigated, with most studies focusing on the relationship between poor motor skills and wellbeing [2]. Poor motor skills have been associated with fewer playmates, fewer spare-time activities, less time spent engaged in social activities and symptoms of anxiety and depression [2]. Motor skills are further seen as an important foundation for participation in physical activities [3] and level of physical activity [1], which has been linked to mental health, wellbeing and fewer emotional and social problems [2-5]. Studies of physical activity or exercise programs indicate improvements in mood, prevention of depression and reduction in depression and anxiety symptoms [4]. The importance of regular motor skills training in preschool children in terms of wellbeing has, to our knowledge, not been investigated previously. In general, there is a need for studies with large sample sizes that follow development for a longer period of time in order to investigate both the short-term and the potential long-term effects of a motor skills intervention on child wellbeing from a developmental perspective [2,4].

Wellbeing is a multi-faceted concept involving physical, psychological, cognitive, social and economic domains. Wellbeing is influenced not just by contextual and individual factors in these domains but also the child’s perception of these factors [5,6]. The motor skills intervention as described in the main body of the application is directed towards improvement in motor skills, but as the intervention is integrated with the preschool curriculum, the intervention may also influence peer interactions and staff-child interactions, both in the introduced motor skills activities and in other more familiar activities. Similarly, the children may experience new opportunities for participation in activities in preschool with the possibility of changes in self-perception.

A prophylactic strategy focusing on improving motor skills activities in preschool children may therefore have the potential to improve the current and future physical, psychological and social wellbeing and their associated health benefits. Based on previous research on poor motor skills and wellbeing, the effect of motor skills training may be stronger for children who are initially characterized by poor motor skills.

### Objectives to be explored in the RCT

The objective is to estimate the effect of a motor skills intervention on wellbeing.

### Objectives to be explored in the cohort study*

- 1. To establish potential patterns of development of psychological wellbeing from three to fifteen years of age.
  2. To determine the influence of motor skills and physical activity in preschool on psychological well-being in adolescence.

** long-term follow-up (12 years)*

### Variables

*Predictors:*

- Motor skills as described in the main application
- Physical activity as described in WP 4

*Outcomes:*

Psychological wellbeing will be measured using the Strengths and Difficulties Questionnaire (SDQ) [7- 9]. The SDQ is a brief questionnaire measuring child and adolescent psychological wellbeing. The SDQ includes 25 items, divided into 5 scales (5 items each): 1) emotional symptoms, 2) conduct problems,

3) hyperactivity/inattention, 4) peer relationship problems and 5) pro-social behavior. The SDQ further includes an impact supplement that asks if the registered behavior is considered a problem and if overall distress, social impairment, burden and chronicity are experienced. The SDQ is widely used to screen for mental health difficulties and as an outcome measure of psychological wellbeing both in clinical practice and in research. A total difficulties score is generated by adding scales 1-4 together and has been found to function as a dimensional measure of child mental health [10]. The questionnaire is available for parent and teacher reporting of children in four different age groups: toddlers (age 2 to 4 years), preschool children (age 5 to 6 years), school children (age 4 to 10 years) and adolescents (age 11 to 17 years). A self-report version is available for adolescents (age 11 to 17 years) [8]. All questionnaires are available in Danish and normative data from Denmark is available for children aged 5 to 12 years [11].

In the RCT, parental and staff reporting will be sampled. The use of multiple informants is commonly recommended to provide the most complete picture of the child’s psychological wellbeing and mental health, as parents and teachers observe children in different contexts, where different behaviors may be exhibited by the child [12]. For the cohort study, the self-report questionnaire is added when the children are aged 11 years or older. The SDQ will be administered electronically to parents and preschool staff twice yearly during preschool and to parents and teachers annually throughout the school years, with the addition of the self-report questionnaire when the child is 11 years of age and older.

The SDQ is expected to take around 10 minutes to complete.

Wellbeing is further measured using the KINDLR [13]. The KINDLR is a generic instrument for assessing health-related quality of life in children and adolescents (age 3 to 17 years). The KINDLR consists of six subdomains, each with four items. In this study, the subscales on physical wellbeing, emotional wellbeing and self-esteem are included (total of 12 items). During preschool, the KiddyKINDL-parents (age 3 to 6 years old) are used. Later, respective of age, parents’ report on the KiddyKINDL or the Kid- kiddoKINDL (age 7 to 17 years old), and children and adolescents report on the KidKINDL (age 7 to 13 years old) or the KiddoKINDL (age 14 to 17 years of age). Danish versions for children and adolescents age 7-17 are freely available. A Danish version of the KiddyKINDL- parents will be made following the instructions provided by the authors. The KINDLR will be administered at baseline and at follow-up as specified in the main protocol. The 12 KINDLR items are expected to take around 5 minutes to complete

The primary outcomes used for analyses in the RCT will be:

- Parent-reported psychological wellbeing, total difficulties score

Secondary outcomes used for analyses in the RCT will be:

- Staff-reported psychological wellbeing, total difficulties score
- Parent-reported wellbeing, total of physical and emotional subscale
- Parent-reported self-esteem

In the cohort study, the following outcomes will be added.

- Self-reported psychological wellbeing, total difficulties score*
- Self-reported wellbeing, total of physical and emotional subscale**
- Self-reported self-esteem**

* For children aged 11 years and older

** For children aged 7 years and older

### Covariates

The following covariates will be accounted for when analyzing the results: sex, age, socioeconomic status, estimated general intelligence (WP7), executive function (WP7) and early developmental difficulties as described in WP8.

### Length of follow-up

For the RCT the follow-up period will be 6 to 30 months, which covers the period from the beginning of the intervention until the last measurement before the child leaves preschool (see Figure 1 in the main protocol). For the cohort study, the duration of the follow up will be 12 years, which covers three years in preschool and nine years in school.

### Analyses

For the RCT study, multilevel linear regression models will be constructed to assess the effectiveness of the intervention on psychological wellbeing. The baseline value of the outcome variable, time since baseline, intervention group and any imbalanced covariates will be added to the model along with preschool and socioeconomic status of the area as random effects. The interaction between preschool and intervention group will be tested. The assumptions for the models will be checked. Similar multilevel linear regression models will be constructed for both the child’s physical and emotional wellbeing and self-esteem. Measures for both groups at baseline and at following points of data collection will be further analyzed using linear mixed effects models (random coefficient models and multilevel models).

The above analyses will be repeated on the children who were considered to have a ‘poor’ level of motor skills at baseline.

In addition to the WP1 analysis of the covariates, an analysis will be conducted to determine if the children who dropped out of the study were different from those who remained, based on the baseline values of the outcome variables.

For the cohort study, longitudinal latent class analysis and latent class growth analysis will be conducted to describe the developmental patterns of wellbeing.

### Perspectives

Increased knowledge of the role of motor skills in the development of wellbeing and insight into the protective potential of early motor skills training could aid in both the future development of high quality preschool care and more specifically target interventions for children with poor motor skills.

### Primary responsibility

Mette Elmose, Assistant Professor, Department of Psychology, University of Southern Denmark.

### References

1. Lubans DR, Morgan PJ, Cliff DP, Barnett LM, Okley AD. Fundamental Movement Skills in Children and Adolescents. Review of Associated Health Benefits. Sports Med. 2010;40(12):1019-1035.
2. Viholainen H, Aro T, Purtsi J. Tolvanen A. Cantell M. Adolescents’ school-related self-concept mediates motor skills and psychosocial well-being. Brit J Educ Psychol. 2014;84:268-280.
3. Soref B, Ratzon NZ, Rosenberg L, Leitner T, Jarus T, Bart O. Personal and environmental pathways to participation in young children with and without mild motor disabilities. Child Care Hlth Dev. 2011;38(4):561-571.doi:10.1111/j.1365-2214.2011.01295.x
4. Penedo FJ, Dahn JR. Exercise and well-being: a review of mental and physical health benefits associated with physical activity. Curr Opin Psychiatry. 2005;18:189-193.
5. Emerson JA, Williams DM. The Multifaceted Relationship Between Physical Activity and Affect. Soc Personal Psychol Compass. 2015 Aug;9(8):419–433.doi: 10.1111/spc3.12190
6. Pollard EL, Lee PD. Child Well-being: A Systematic Review of the Literature. Soc Indic Res. 2003 Jan;61(1):59-78.
7. Goodman R. The Strengths and Difficulties Questionnaire: A Research Note. J Child Psychol Psychiatry 1997;38:581-586.
8. Goodman R, Meltzer H, Bailey V. The Strengths and Difficulties Questionnaire: A pilot study on the validity of the self-report version. Eur Child Adoles Psy*.*1998;7:125-130.
9. Goodman R. The extended version of the Strengths and Difficulties Questionnaire as a guide to child psychiatric caseness and consequent burden. J Child Psychol Psychiatry. 1999;40:791-801.
10. Goodman A, Goodman R. Strengths and difficulties questionnaire as a dimensional measure of child mental health. J Am Acad Child Adolesc Psychiatry. 2009;48:400-403.
11. Niclasen J, Teasdale TW, Andersen AN, Skovgaard AM, Elberling H, Obel C. Psychometric Properties of the Danish Strength and Difficulties Questionnaire: The SDQ Assessed for More than 70,000 Raters in Four Different Cohorts. PLoS ONE. 2012 Feb;2(7):e32025. doi:10.1371/journal.pone.0032025
12. Stone LL, Rutger RO, Engels CME, Vermulst AA, Janssens JMAM. Psychometric Properties of the Parent and Teacher Versions of the Strengths and Difficulties Questionnaire for 4- to 12-Year-Olds: A Review. Clin Child Fam Psychol Rev. 2010 Sep;13(3):254–274. doi:10.1007/s10567-010-0071-2
13. Ravens-Sieberer U, Bullinger M. Assessing health-related quality of life in chronically ill children with the German KINDL: first psychometric and content analytical results. Quality of Life Research. 1998;7(5):399- 407.

**WP 7: Motor Skills and Effects on Executive Functions**

### Background

It is well established that cognitive ability is a powerful determinant of success in many aspects of social and economic life (e.g. Cunha and Heckman 2007; Duckworth et al. 2011). However, more recent research suggests that non-cognitive abilities (e.g. conscientiousness, grit, and self-control) in early life are at least as important as cognitive abilities for later outcomes such as school readiness and educational careers (e.g. Duckworth et al. 2011). Based on recent literature on child development, Cunha and Heckman (2007) found that non-cognitive abilities have direct effects on wages (controlled for schooling), teenage pregnancy, smoking, crime, performance on achievement tests and many other aspects of social and economic life.

The concept of ‘non-cognitive skills’ has been used broadly about skills other than academic skills such as literacy and numeracy. We focus on the link between motor skills and one important aspect of non- academic skills: executive functions, which cover both working memory (which is related to IQ) and inhibitory control, which includes inhibition of thoughts, attention and behaviour (Almlund et al. 2011; Diamond 2014a). We focus on executive functions because most prior work on the non-academic effects of motor skills interventions focuses on executive functions. Within executive functions, we focus on inhibitory control because it is related to concepts such as conscientiousness and self-control that are highly predictive of many long-term outcomes (see Almlund et al. 2011 for a comprehensive review of this literature). Among the big five personality traits, conscientiousness is the strongest predictor of long-term outcomes (Heckman and Kautz 2012). It has been defined as “consistency of action resulting from deliberate volition, or will” (Almlund et al. 2011, p. 10). Conscientiousness is a measure of whether the child manages to accomplish more work, makes an effort, pushes her/himself hard to succeed, is good at organizing, is good at avoiding distractions, does not put off unpleasant tasks, makes careful decisions, is goal-oriented, does not give up easily, remains calm under pressure and the like (MacCann et al. 2009).

Other related traits are grit and self-control (Almlund et al. 2011). Self-control is also highly predictive of a broad range of long-term outcomes even after controlling for academic skills (Moffitt et al. 2013). Although operational definitions of self-control vary widely, Duckworth and Kern (2011) argue that a central commonality is: *“(…) the idea of voluntary self-governance in the service of personally valued goals and standards”* and the *“(…) capacity of altering one’s own responses, especially to bring them into line with standards such as ideals, values, morals, and social expectations, and to support the pursuit of long-term goals”* (Duckworth and Kern 2011, p. 260).

Importantly, recent studies suggest that non-cognitive skills are not innate, but malleable and teachable (Cunha and Heckman 2007; Diamond 2014b). Yet, skills acquired in one period persist into future periods – and skills produced in one period raise the productivity of investments in subsequent periods.

Research implies that better fine and gross motor skills in themselves improve attention, working memory, disciplined action, joy, pride, and social bonding – besides the more obvious improvement of physical wellbeing and fitness (Bugge et al. 2015; Diamond 2014b; Diamond and Lee 2011). The relationship between motor skills and non-cognitive abilities has mainly been studied in small-scale studies in the USA. Despite promising initial results (e.g., Diamond and Lee 2011), the research in this area is still in its early stages with little systematic evidence. In our review of recent literature, we found only seven studies

analyzing the benefits of motor skills interventions (see Table 1 for an overview of the seven studies)1. Of these studies, only one (i.e., Lakes et al. 2004) studies preschool children and most of the studies include small samples of children.

### Table 1: Overview of previous physical studies (RCTs) with self-control related measures as one of their outcome measures

| **Study** | **Type of physical intervention. Type of motor skill in primary focus (fine, gross or balance). Age group. Country.** | **Main results regarding self-control** |
| --- | --- | --- |
| **Sørensen, Mona Have (2015): ”Aktiv Matematik i 1. Klasse. Et skolerandomiseret kontrolleret studie.” in *Rapport for ”Forsøg med Læring i Bevægelse”,* edited by Anna Bugge and Karsten Froberg, Syddansk Universitet, Institut for Idræt og Biomekanik.** | Physical activity in math lessons. Gross motor skills (an active break every 20 minutes throughout the math lesson, making the children able to better concentrate on math after raising the activity level). 6-7-year olds.  Denmark. | Small benefits of exercise on boys’ executive function. |
| **Davis, Catherine L. et al. (2011): *Exercise Improves Executive Function and Achievement and Alters Brain Activation in Overweight Children: A Randomized Controlled Trial. Health Psychol.,* 30: 91-98.** | Aerobic exercises selected based on ease of comprehension, fun, and eliciting intermittent vigorous movement.  Gross motor skills (focus on intensity through activities such as running, basketball, and soccer). 7- 11-year olds. USA. | Benefits of exercise on executive function. |
| **Kamijo, Keita et al. (2011): *The effects of an afterschool physical activity program on working memory in preadolescent children. Dev. Sci.,* vol. 14.** | Physical activity based on improvement of especially cardiorespiratory fitness through engagement in a variety of activities. Gross motor skills (cardiorespiratory fitness). 7-9 year olds.  USA. | Benefits of exercise on Sternberg task performance. |

1 Most of these studies were found in systematic reviews about self-control interventions for children and youth up till 17 years of age (Oxford 2013; Diamond and Lee 2011; Piquero et al. 2010). Still, some of them were so new that they have not been reported in systematic reviews.

| **Weis, Robert, and Toolis, Erin E. (2009): *Evaluation of a voluntary military-style residential treatment program for youths with conduct problems: 6- and 36-month outcomes*. *Psychological Services,* 6(2):139- 153** | Boot camp training consisting of hard military training and school. Gross motor skills.16-18-year olds. USA. | Six months after treatment, those who completed showed fewer externalizing problems, greater adaptive skills, and better behavioural outcomes. Results were not maintained at 36- month follow-up. |
| --- | --- | --- |
| **Lakes, Kimberley D., and William T. Hoyt (2004): *Promoting self-regulation through school-based martial arts training. Applied Developmental Psychology,* 25: 283-302.** | Tae Kwon Do training. Balance and fine motor skills. Kindergarten through Grade 5. USA. | Benefits of exercise in areas of cognitive and affective self-regulation, prosocial behaviour, and classroom conduct. |
| **Zivin, Gail; Nimr R Hassan, Geraldine F. DePaula, Daniel A. Monti, Carmen Harlan, Kashfia D. Hossain, and Ksai Patterson (2001): *An effective approach to violence prevention: Traditional martial arts in middle school. Adolescence,* 36(143): 443-**  **459** | Koga Ha Kosho Shorei Ryu Kempo training. Balance and fine motor skills. Boys in middle school. USA. | Improvement in self- control appeared. |
| **Manjunath, NK, and S Telles (2001): *Improved performance in the Tower of London test following yoga. Indian J Psysiol Pharmacol,* 45: 351-354.** | Yoga training. Balance and fine motor skills. 10- 13-year old girls. India. | Benefits of exercise on executive function, with improvements most evident when executive function demands were greatest. |
| **Sources:** The American systematic Campbell review from 2010 (Piquero et al. 2010) on self-control interventions for children under age 10 for improving self-control, delinquency and problem behaviours.  - **The American systematic review made by Diamond and Lee in 2011 (Diamond and Lee 2011) on interventions shown to aid executive function development in 4–12-year olds.** - **The Danish systematic Oxford review from 2013 (Oxford 2013) on methods that can improve self-control in 7-17-year olds.** - **An RCT from the recently published Danish report from Southern University (Bugge et al. 2015).** | | |

**Objectives**

1. To investigate whether a motor skills intervention improves the preschool children’s executive functioning, especially in terms of self-control and inhibitory control.
2. To investigate the effect of a motor skills intervention on academic performance.

### Variables

*Predictors:*

Gross and fine motor skills as described in the main protocol.

*Outcomes:*

The Preschool Self-Regulation Assessment Battery (PSRA)

The PSRA was designed to assess self-regulation in emotional, attentional, and behavioural domains by using a brief, structured battery of tasks in conjunction with a global report of children’s behaviour. This test can easily be translated into Danish. It includes balance beam and pencil tapping. Age span: 3-5 years. Time consumption: Four brief tasks; 8-10 minutes (Smith-Donald et al. 2007).

Academic performance

Academic performance will be assessed based on the Grade 9 school examinations school grades and the Danish National Test. The Danish National Tests are described in work package 5. Grades from all six subjects in the Danish National Test from Grade 2 to Grade 8 will be used.

### Covariates,

The following covariates will be accounted for when analysing the results: sex, age, socioeconomic status, baseline estimated general intelligence and child wellbeing.

Reynolds Intellectual Screening Test (RIST)

RIST is developed to provide a quick and reliable measure and is used to measure an estimated general intelligence at baseline. The RIST consist of two of the subtests from Reynolds Intellectual Assessment Scale (RIAS). The four RIAS subtests to measure general intelligence include two nonverbal tests and two verbal tests. The RIST consists of one verbal test (Guess what) and one nonverbal test (Odd item out). The test is appropriate for individuals ages 3 to 94. The median reliability coefficient for the RIST Index is .95 and the test-retest reliability is .84 (Reynolds & Kamphaus, 2003). RIAS has been found to correlate highly with the other assessments of general intelligence (Edwards & Paulin, 2007) and there is a high correlation between RIST and RIAS (Reynolds & Kamphaus, 2003).

### Length of follow-up,

The duration of the follow-up period is six months to three years (preschool period)

### Analyses

Simple univariate statistics will be calculated to describe the outcomes and covariates by school.

Multilevel linear regression models will be constructed to assess the effectiveness of the intervention on executive functioning. The baseline value of the outcome variable, time since baseline, intervention group and the covariates of sex, age, estimated general intelligence, socioeconomic status of the household and child well-being at baseline will be added to the model along with school and socioeconomic status of the area as random effects. The interaction between school and intervention group will be tested. The assumptions for the models will be checked.

Similarly multilevel linear regression models will be constructed to assess the effectiveness of the intervention on academic performance as measured by the Grade 9 examination grades. The baseline value of the outcome variable, time since baseline, intervention group and the covariates of sex, age, estimated general intelligence, socioeconomic status of the household and child well-being at baseline will be added to the model along with school and socioeconomic status of the area as random effects. The interaction between school and intervention group will be tested. The assumptions for the models will be checked.

### Primary responsibility

Simon Calmar Andersen, Professor, Department of Political Science and Government, Aarhus University and part of the center management at TrygFonden’s Centre for Child Research, Aarhus University.

### References

Almlund, M., A. Duckworth, J. Heckman and T. Kautz (2011): “Personality Psychology and Economics,” (with). In *Handbook of the Economics of Education*, edited by E. Hanushek, S. Machin, and L. Woessman, pp. 1-181, Elsevier: Amsterdam.

Bugge, Anna, and Karsten Froberg (ed.) (2015): *Rapport for Forsøg med Læring i Bevægelse. Syddansk Universitet*, Institut for Idræt og Biomekanik.

Cunha, Flavio, and James Heckman (2007): *The Technology of Skill Formation. NBER Working Paper* 12840.

Davis, Catherine L., Phillip D. Tomporowski, Jennifer E. McDowell, Benjamin P. Austin, and Patricia H. Miller (2011): *Exercise improves executive function and achievement and alters brain activation in overweight children: A randomized, controlled trial. Health Psychology,* 30(1): 91-98.

Diamond, Adele (2014a): *Understanding Executive Functions: What Helps or Hinders Them and How Executive Functions and Language Development Mutually Support One Another. The International Dyslexia Association.*

Diamond, Adele (2014b): “Want to Optimize Executive Functions and Academic Outcomes? Simple, Just Nourish the Human Spirit” In *Minnesota Symposia on Child Psychology Developing Cognitive Control Processes: Mechanisms, Implications, and Interventions*, Philip David Zelazo and Maria D. Sera. Wiley, volume 37.

Diamond, Adele, and Kathleen Lee (2011): *Interventions Shown to Aid Executive Function Devel-opment in Children 4 to 12 Years Old. Science,* 333: 959-964.

Duckworth, Angela Lee, James J. Heckman, and Tim D. Kautze (2011): *Personality Psychology and Economics. NBER Working Paper Series* 16822.

Duckworth, Angela Lee, and Margaret L. Kern (2011): *A meta-analysis of convergent validity of self-control measures. Journal of Research in Personality*, 45: 259-268.

Edwards, Oliver, W. & Paulin, Rachel, V. (2007): Referred students’ performance on the Reynolds Intellectual Assessment Scales and the Wechsler Intelligence Scale for Children- Fourth Edition. Journal of Psychoeducational Assessment. Vo. 25(4): 334-340.

Heckman, James J. and Tim Kautz (2012): *Hard evidence on soft skills. Labour Economics,* Elsevier, 19(4): 451-464.

Kamijo, Keita, Matthew B. Pontifex, Keivin C. O’Leary, Mark R Scudder, Chien-Ting Wu, Darla M. Castelli, and Charles H. Hillman (2011): *The effects of an afterschool physical activity program on working memory in preadolescent children. Dev. Sci.,* vol. 14.

Lakes, Kimberley D., and William T. Hoyt (2004): *Promoting self-regulation through school-based martial arts training. Applied Developmental Psychology,* 25: 283-302.

MacCann, Carolyn; Angela Lee Duckworth; and Richard D. Roberts (2009): Empirical identification of the major facets of Conscientiousness. *Learning and Individual Differences.*

Manjunath, N. K., and S. Telles (2001): *Improved performance in the Tower of London test following yoga.*

*Indian J Psysiol Pharmacol,* 45: 351-354.

Moffitt, Terrie E., Richie Poulton, and Avshalom Caspi (2013): *Lifelong Impact of Early Self-Control.*

*Childhood self-disciplin predicts adult quality of life. American Scientist,* vol. 101.

Oxford Research (2013): *Systematisk kortlægning af metoder der kan styrke børn og unges selvkontrol.*

Udgivet af Oxford Research for Det Kriminalpræventive Råd.

Piquero, Alex R., Wesley G. Jennings, David P. Farrington (2010): *Self-control interventions for children under age 10 for improving self-control and delinquency and problem behaviors. Campbell Systematic Reviews,* 2010: 2.

Reynolds, Randy & Kamphaus, Cecil (2003): *Reynolds Intellectual Assessment Scales and Reynolds Intellectual Screening Test Professional Manual*, Psychological Assessment Resources (2003)

Rueda, Rosario M., Jin Fan, Bruce D. McCandlies, Jessica D. Halparin, Dana B. Gruber, Lisha Pappert Lercari, and Michael I. Posner (2004): Development of attention networks in childhood. *Neuropsychologia* 42, 1020-1040.

Rueda, Rosario M., Mary K. Rothbart, Bruce D. McCandlies, Lisa Saccomanno, and Michael L. Posner (2005): *Training, maturation, and genetic influences on the development of executive attention. PNAS,* 41: 14931-14936.

Smith-Donald, Radiah, C. Cybele Raver, Tiffany Hayes, and Breeze Richardson (2007): *Preliminary construct and concurrent validity of the Preschool Self-regulation Assessment (PSRA) for field-based research. Early Childhood Research Quarterly,* 22: 173-187.

Sørensen, Mona Have (2015): ”Aktiv Matematik i 1. Klasse. Et skolerandomiseret kontrolleret studie” In *Rapport for Forsøg med Læring i Bevægelse*, edited by Anna Bugge and Karsten Froberg, Syddansk Universitet, Institut for Idræt og Biomekanik.

Weis, Robert, and Elin E. Toolis (2009): *Evaluation of a voluntary military-style residential treatment program for youths with conduct problems: 6- and 36-month outcomes. Psychological Services,* 6(2): 139-153.

Zivin, Gail, Nimr. R. Hassan, Geraldine F. DePaula, Daniel A. Monti, Carmen Harlan, Kashfia D. Hossain, and Ksai Patterson (2001): *An effective approach to violence prevention: Traditional martial arts in middle school. Adolescence,* 36(143): 443-459.

# WP 8: Associations between motor skills, physical activity, cognition, psychological wellbeing and language development for children at developmental risk.

**Background**

Developmental difficulties, such as, but not limited to, Intellectual Disability (ID), Downs syndrome (DS), Cerbral Palsy (CP), Autism Spectrum Disorders (ASD), and Attention Deficit Hyperactivity Disorder (ADHD), are associated with motor performance difficulties. Developmental difficulties are further associated with different patterns of participation in both daily physical and play activities [1,2].

Motor skill difficulties may in themselves function as a barrier to physical activity. However, other factors related to developmental difficulties have been found to impose further psychological, practical, social and societal barriers to participation [1,3]. Examples of these barriers are related social difficulties, behavioral problems, exclusion, strain on family or staff, other constraints on family time and resources, a higher need for adult supervision as well as lack of skills to include the children [1,2,3]. The number of parent-reported barriers to physical activities have been directly linked to both hours spent in physical activity as well as total screen time [3]. The patterns of participation in daily physical and play activities influence the child’s opportunities to develop both motor and social skills and over time further reduce the child’s opportunities to gain positive experiences with these activities. Possible facilitators that may support physical participation for children with developmental difficulties are currently being identified in practice [4] and programs adapted to children and adolescents with developmental difficulties are being developed, one example in Denmark being FOKUS [5]. In FOKUS, shooting sports are made available to children and adolescents with ADHD and similar developmental difficulties through collaboration between the Danish Society for Gymnastics, municipalities and schools [5]. The aim is to increase the opportunities for participation in organized sports activities. Current research evidence suggests that physical activity may be an effective non-pharmaceutical intervention approach for children with developmental disorders [1] and may help improve sleep quality [6], reduce stereotypical behaviors [7] and reduce behavioral problems [8]. Understanding the extent to which physical activity alters developmental trajectories of mental health is of increasing importance [1].

A motor skills intervention may be of particular importance for children at early developmental risk and, if effective, may be associated with larger overall improvements.

### Objectives to be explored in the RCT

1. To estimate the effect of a motor skills intervention on motor skills, physical activity, cognition, language development and wellbeing for a subgroup of children at early developmental risk.
2. To look at specific barriers to, and facilitators of, participation of children at early developmental risk in the motor skills program.

### Objectives to be explored in the cohort study

1. To establish potential patterns of development of motor skills, physical activity, cognition, language development and wellbeing from three to fifteen years of age for a subgroup of children at early developmental risk.*
2. To determine the influence of motor skills, physical activity, verbal development, social-emotional development, early developmental concerns and wellbeing on developmental difficulties in adolescence.*

** long-term follow-up (12 years)*

### Variables

A subgroup of children will be included in the sub-group of children at early developmental risk based on the following information and measures:

- - Information on early medical and psychiatric diagnoses is collected from parents at baseline and at the last follow-up before starting school. Afterwards, this information is collected yearly during the school years.
  - Strengths and Difficulties Questionnaire (SDQ) is completed as described in WP 6. Computerized algorithms have been developed to estimate the likelihood of a child’s having a psychiatric diagnosis based on symptom and impact scores derived from the SDQs [9]. These algorithms are used to generate likelihood ratings (either: unlikely, possible or probable) for four broad categories of disorder: conduct disorders, emotional disorders, hyperactivity disorders and any psychiatric disorder. The algorithm has been found to correctly identify 81-91% of children who definitely had the indicated clinical diagnosis and is described as sufficiently accurate and robust

to be of practical value [9]. In this study, a probable rating of any of the four categories of disorders is seen as an indicator of a probable developmental disorder and henceforth as early developmental risk.

Further, information on the children’s early social-emotional development and the staff observations of the children’s wellbeing will be collected in the Municipality of Svendborg in the same period. Staff evaluations of child wellbeing are systematically collected twice yearly following written guidelines for the evaluation [10]. Part of this evaluation is the discussion of four domains of child wellbeing (psychological and emotional, cognitive, social and physical). Based on these discussions, a rating of thriving, cause for concern or strong concern is made for the overall wellbeing of each child. These registrations will be available for the study.

Thus, the child is perceived as being at early developmental risk if:

- - Parents report having received a formal child psychiatric diagnosis for the child prior to or during the project period.
  - Parents report having received a formal medical diagnosis for the child, during the project period. The focus is on pervasive diagnosis such as deafness, CP and DS which is known to influence development more pervasively.
  - The SDQ algorithm indicates the likelihood of a psychiatric disorder on the basis of either the completed parent or staff questionnaire.
  - Staff rate high developmental concern in the RCT period.

The child is perceived as having developmental difficulties (as an outcome) if:

- Parents report having received a formal child psychiatric diagnosis for the child.
- Parents report having received a developmentally pervasive medical diagnosis for the child.
- The SDQ algorithm indicates the likelihood of a psychiatric disorder on the basis of the completed child, parent or teacher questionnaire at the last point of data collection.

This broad inclusion and practical demarcation of developmental difficulties is used in order to get a broader understanding of the developmental mechanisms related to children at developmental risk.

The following covariates will be accounted for when analyzing the results: sex, age, socioeconomic status as well as estimated general intelligence and executive functions as described in WP 7.

Barriers and facilitators:

Barriers to, and facilitators of, implementation in relation to children at early developmental risk are investigated using observations of participation from the perspective of the child [13]. Child participation will be observed in different preschools, both in activities defined as part of the motor skills intervention as well as in a number of other activities. Further, semi-structured qualitative interviews [14] with staff in the preschools will be included to strengthen the understanding of potential barriers and facilitators associated with children at early developmental risk.

### Length of follow-up

The duration of the follow-up period will be 12 years, which cover three years in preschool and nine years in school.

### Analyses

The analytic strategies described in the other WPs will be repeated on the subgroup of children who are identified as having early developmental difficulties.

For the cohort study, longitudinal latent class analysis and latent class growth analysis will be conducted to describe the patterns of developmental difficulties.

To describe barriers to, and facilitators of, implementation of the motor skills intervention for children at early developmental risk, observations and interviews are analyzed using a phenomenological analytic approach [14] to understand the participation of the child.

### Perspectives

The project will bring new insight into the relevance of motor skills and physical activity for children at developmental risk. Understanding how motor skills and physical activity are associated with trajectories of developmental difficulties and the extent to which early intervention might affect these trajectories is of great importance for developing efficient strategies for prevention or intervention associated with developmental difficulties.

### Primary responsibility

Mette Elmose, Assistant Professor, Department of Psychology, University of Southern Denmark.

### References

1. Pontifex MB, Fine JG, da Cruz K, Parks AC, Smith AL. VI. The role of physical activity in reducing barriers to learning in children with developmental disorders. Monogr Soc Res Child Dev. 2014 Dec;79(4):93-118. doi: 10.1111/mono.12132.
2. Memari AH, Panahi N, Ranjbar E, Moshayedi P, Shafiei M, Kordiet R, Ziaee,V. Children with autism spectrum disorder and patterns of participation in daily physical and play activities. Neurology Research International. 2015. Available from: [http://dx.doi.org/10.1155/2015/531906.](http://dx.doi.org/10.1155/2015/531906)
3. Must A, Phillips S, Curtin C, Bandini LG. Barriers to physical activity in children with autism spectrum disorders: relationships to physical activity and screen time. J Phy Act Health. 2015;12:529-534. Available from [http://dx.doi.org/10.1123/jpah.2013-0271.](http://dx.doi.org/10.1123/jpah.2013-0271)
4. Bendtholm A. Idræt flytter grænser – for børn med autism og ADHD. 1st ed. Handicapidrættens

Videnscenter.c2013.

1. Veel H. Skydeidræt er gavlingt for børn med ADHD DGI [Internet]. 22015 Jan 15 [cited 2015 Aug 25]. Available from: <http://www.dgi.dk/om/samarbejd-med-dgi/projekter/fokus-ro-og-koncentration-gennem-> skydeidraet/seneste-nyt-fra-fokus/skydeidraet-er-gavnligt-for-boern-med-adhd.
2. Wachob D, Lorenzi DG. Brief Report: Influence of Physical Activity on Sleep Quality in Children with Autism. J Autism Dev Disord. 2015;45:2641-2646. doi:10.1007/s10803-015-2424-7
3. Petrus C, Adamson SR, Block L, Einarson SJ,Sharifnejad M, Harris SR. Effects of Exercise Interventions on Stereotypic Behaviours in Children with Autism Spectrum Disorder. Physiother Can. 2008;60:134-145.
4. Verret C, Guay m, Berthiaume C, Gardiner P, Béliveau L. A physical activity program improves behavior and cognitive functions in children with ADHD: An exploratory study. J Atten Disord. 2012;161):71- 80.doi:10.1177/1087054710379735
5. Goodman R, Renfrew D, Mullick M. Predicting type of psychiatric disorder from strengths and difficulties questionnaire (SDQ) scores in child mental health clinics in London and Dhaka. Eur Child Adoles Psy. 2000;9:129-134.
6. Mehlbye J, Andersen J. (eds). Tidlig opsporing af børn I en social udsat position. Idekatalog [Internet]. Kora. Det Nationale Institut for Kommuners og Regioners Analyse og Forskning. 2012 [cited 2015 August 25]. Available from: [http://www.kora.dk/media/333763/5003_idekatalog_opsporingsmodel.pdf.](http://www.kora.dk/media/333763/5003_idekatalog_opsporingsmodel.pdf)
7. Squires J, Bricher D, Waffell M, Funk K, Clifford J, Hoselton R. Social-Emotional Assessment/Evaluation Measure (SEAMTM), Research Edition. Brookes Publishing co. c2014.
8. Pontoppidan M, Niss NK. Instrumenter til at male små børns trivsel [Internet]. SFI – Det Nationale Forskningscenter for Velfærd. 2014 [cited 2015 August 25]. e-ISBN: 978-87-7119-252-0. Available from: [http://sfi.dk/rapportoplysninger-4681.aspx?Action=1&NewsId=4443&PID=9267](http://sfi.dk/rapportoplysninger-4681.aspx?Action=1&amp;NewsId=4443&amp;PID=9267)
9. Pedersen M, Klitmøller J, Nielsen K. Deltagerobservation. 1st ed. Hans Reitzel. c2012.
10. Kvale S, Brinkmann S. Interview – Det kvalitative forskningsinterview som håndværk. 3rd ed. Hans Reitzel. c2015.
