# Supplementary material for: Influence of motor skills training on children’s development evaluated in the Motor skills in PreSchool (MiPS) study-DK: study protocol for a randomized controlled trial, nested in a cohort study
Source: Trials. 2017 Aug 29;18:400. doi: 10.1186/s13063-017-2143-9 (PMC5576290; doi:10.1186/s13063-017-2143-9)
Supplement: Supplementary file 2 — Svendborg Kommune/Municipality of Svendborg. (DOCX 71 kb) [file 13063_2017_2143_MOESM2_ESM.docx]

**Additional file 2 Svendborg Kommune /**

**Municipality of Svendborg**

2a: Project organization within the municipality

2b: Concept description

#### Project organization and staffing

The project is organized under the auspices of the Svendborg Municipality with a steering committee and three working groups.

The first task of the steering committee will be to approve a project description.

#### Steering committee

- Birgit Lindberg, daycare manager (chair person). o Morten Refskou, head of culture (ad hoc)
- Anne Højmark, head of health
- Kirsten Søndergaard, daycare director o Lise Hestbæk, SDU
- Linda Pedersen, coordinator, Sport Study Svenborg/Sport & Uddannelse o Mette Skov Hansen, project manager, The Svendborg Project’s

**The working Group ‘Concept development’**

- - - Has the responsibility to develop the learning environments we must have, and put together an activity programme for movement, motor skills and body awareness. The group must also deal with how best to incorporate the digital world.
    - The working group consists of:
- Liselotte Nielsen, educational team leader, Dagplejen SydØst
- Michael Axelsen, educational team leader, Sundet
- Dorte Møller Pedersen, motor educator, Vesterlunden-Nordenvinden
- Mette Sorensen, physiotherapist, Svendborg Kommune
- Kirsten Søndergaard, daycare director
- Louise Madsen, health consultant
- Lisbeth Runge, RICH, SDU
- Jacob Kramer, Svendborg Gymnastikforening
- Mette Sorang Kjær, Hans Christian Andersen’s Børnehospital (ad hoc)
- Mette Skov Hansen, Svendborgprojektet
- Linda Pedersen, coordinator, Sport & Education

**The working group "Research"**

- - - Has the responsibility to plan the implemention and coordination of data collection and discuss and confirm the design of the research

The working group consists of:

- Lise Hestbæk, associate professor, Institut for Idræt og Biomekanik, SDU
- Henrik Hein Lauridsen, Associate Professor, Institut for Idræt og Biomekanik, SDU
  - - Marianne Sigvardt, educational team leader, Egebjerg
    - Jesper Eilsø, health consultant
    - Dorte Røgild, healthcare nurse
    - Susanne Andersen, educational team leader, Østerdalen
    - Kirsten Søndergaard, day care director
    - Anja Kastrup Jensen, associate professor of psychology and cand. Idræt & Psykologi, UCL
    - Lone Sorensen, physiotherapy / occupational therapy, Svendborg Kommune
    - 1 from Hans Christian Andersen’s Børnehospital (ad hoc)
    - Mette Skov Hansen, project manager, Svendborgprojektet

**The working group "Competence development"**

- Has the responsibility to plan the training of teachers of young children, their assistants, child care aides and child carers
- The working group consists of:
  - - Anja Kastrup Jensen, associate professor of psychology and cand. Scient i idræt & Psykologi, UCL Pædagoguddannelse
    - Lene Juul Larsen, physiotherapy / occupational therapy, Svendborg Kommune
    - Susanne Andersen, educational team leader, Østerdalen
    - Vera Hansen, motor educator, Sundet
    - Bodil Schjøtt, daycare
    - Lone Tang Jørgensen, Pædagoguddannelse, Svendborg
    - Kirsten Søndergaard, daycare director
    - 1 teacher from Praksisvidencenter Svendborg
    - Mette Skov Hansen, Svendborgprojektet
    - Line Harvesting, coordinator, Praksisvidencenter Svendborg

In addition, there will be various degrees of ad hoc involvement of other relevant stakeholders.

#### Concept for Active Children in Daycare

- healthier, happier, smarter children

#### Vision

In Svendborg, we have a vision - we want:

*Denmark's most active 0-6-year olds - the child and movement in the center.*

#### Mission

The project's vision is obtained through the mission that:

*Daycare services in Svendborg provide learning environments that create the foundation for a healthy life, centered around the body and movement.*

#### Goals

Specifically, the concept's overall objective is:

*To develop learning environments in daycare centres that create more movement, strengthen children's motor skills and increase body awareness among children. This is done through physical activity of high quality.*

#### Background

Physical activity, play, movement and motor development are different sides of the same coin - especially when talking about children aged 0 to 6 years. Physical activity not only has some significant health benefits, but it is also an effective teaching tool.

In order to encourage more physical activity, the Svendborg Municipality has launched the project *Active Children in Daycare* to put even more focus on physical activity, play, movement and motor development amongst children in the Municipality’s daycare centres.

The project is an offshoot of Denmark’s largest sports school project, *The Svendborg Project,* where all children in Grades 0-6 in the Municipality’s schools have six lessons of sport each week. The research in The Svendborg Project has shown that the extra lessons of sport have significant and positive effects on children's health, for example, in relation to obesity and the risk factors associated with lifestyle diseases.

A large part of children's motor development occurs from the age of 0 to 6 years. Therefore, there is great potential to work even more with physical activity in the programmes of daycare centres. The Municipality's daycare centres are already working on motor skills and health, and by launching *Active Children in DayCare*, we will strengthen these efforts and promote children's physical and motor development. This is also important because children with mobility challenges are generally less physically active than other children, and because physical activity is an important parameter in children's health. The Svendborg Project has shown that the children who most need a health boost are also the ones who benefit most from the additional sport in school. Similarly, action taken in daycare centres is supposed to be an effective tool because it helps and boosts *all* children.

*Active Children in Daycare* is thus an early preventive and health-promoting strategy that gives all children in the participating daycare centres an experience of the joy of movement and gives them physical strength for a healthy start in life. At the same time, physical activity is a really effective educational tool that can contribute to children's well-being, development, learning and upbringing- physically, emotionally, socially and cognitively. That is why the project is called *Active Children in Daycare* - *healthier, happier and smarter children!*

#### Definition of Gazelle Children’s House and Gazelle Daycare

The participating children’s houses and daycare centres are referred to as Gazelle Children’s Houses and Gazelle Daycare Centres respectively, and are defined as places where there is a particular focus on physical activity and the use of physical activity as a pedagogical tool. Physical activity is thus used both as an activity with a targeted focus on children’s physical development and as a fun and motivating tool to achieve other learning objectives.

It is important to note that the age span of children in daycare is large. Therefore, the motor challenges and intensity of activities must be adapted to the individual child's level of development. We call it *development-oriented educational sport*.

In addition to this general description are the following specific requirements for the participating Gazelle Children’s Houses and Gazelle Daycare Centres.

#### The five Gazelle leaps

‘Gazelle leaps’ is the name given to the elements that the Gazelle Children’s Houses and Gazelle Daycare Centres must meet:

1. At least four days a week, and preferably five, a minimum of 45-minute adult-initiated and adult-led activities must be carried out, where all children participate. During the week, all bodily skills mentioned below must be challenged.
2. The majority of the planned activities must be carried out outdoors.
3. The body and movement must be used as an educational tool in working with other learning-level themes - called educational sport.
4. All children must have an elevated pulse every day
5. The individual daycare centre must develop learning environments in accordance with the description

*Learning Environments in Active Children in Daycare* - *healthier, happier and smarter children*

#### At least four days a week, and preferably five, a minimum of 45-minute adult-initiated and adult-led activities must be carried out, where all children participate. During the week, the following bodily skills must be challenged:

| Bodily skills | What and how ? (examples) | Why? (examples) |
| --- | --- | --- |
| Motor function | *Gross motor challenges* such as creeping, crawling, running, jumping, hopscotch, jumping, climbing  *Fine motor challenges* such as holding a pencil, handling small objects like beads and construction toys or catching insects  *Coordination Exercises* such as crawling exercises, cross-body movements, "Angels in the Snow", jumping jack and throwing, gripping and kicking exercises. Other examples can be rhythm and dance.  Different dynamic and static *balance exercises* such as walking on a line and standing on one leg. | Gross and fine motor skills are important because they form the basis of many everyday activities and are an important factor for the child's enjoyment of physical activity and thereby also the amount of the child’s physical expression.  Coordination is the foundation for a long series of specific skills such as throwing and catching and many everyday activities such as pouring water into a glass.  A good balance is, amongst other things, important in relation to avoiding falls and injuries, and affects many daily activities such as putting on clothes and shoes. |
| Sensing | Challenges of the following senses:  The *vestibular* sense is stimulated for example by rolling, turning around, doing somersaults and swinging.  The *tactile* sense is stimulated by touch from others, for example, in the form of massage and by touching various materials and objects of different size, shape and temperature.  The *kinaesthetic* sense is stimulated by challenging the body's joints, muscles and tendons in different ways, for example, by bending, stretching and pushing, lifting objects of different weights and by fast and slow movements. | We use all of our senses to collect and process the information and experiences that we give our body and our brain. The senses are thus to control and develop our balance, coordination and motor skills and are thereby important to be able to perform both defined work routines and everyday activities. |
| Relaxation | The children will also experience other types of physical stimulus, namely relaxation and unwinding. It can, for example, be through massage, children’s yoga or similar. | Relaxation is a good counterpart to dynamic activity, which together promote body consciousness in children. At the same time, relaxation helps to create calmer children and fewer conflicts. |

It is important to point out that there is a close link, and also a direct overlap, between the different bodily skills. Therefore, an activity may contain elements of several skills. For example, exercises that stimulate the vestibular sense often also contain elements of balance.

The individual children's houses and daycare centres organize the specific activities themselves. In mixed groups of children of various ages, it is recommended that, where possible, to divide the children up, so that the groups are about the same developmental level. It provides greater opportunity to target the activity to the individual child and less need for differentiation of activities.

#### The majority of the planned activities must be carried out outdoors

Life outdoors generally creates more physical activity and better body awareness among children. This means the children's physique is challenged more because nature encourages and even requires that children crawl, jump, roll or balance.

#### Educational sport: body and movement is used as an educational tool in working with other curriculum themes

Educational sport is the name of an educational approach which uses physical activity as a tool to achieve educational goals that are not necessarily related to children's physical and motor development. Physical activity, play, movement and motor development are inextricably linked when you talk about children in daycare. Both the children's desire to move and the level of physical activity depend very much on how good their motor skills are. In addition, motor skills have a number of other major positive effects, amongst other things, on the child's self-esteem and social competence. Physical activity is thus a teaching tool that can be used to promote physical, mental, social and cognitive development in children. This is due primarily to bodily experiences that are better retained in the mind and the body's subconscious, because they are experienced by several of the body's senses at the same time. When the body is in use, several centers of the brain are awakened, which increases learning. Children who have a good understanding of the body will also be more familiar with their body and have better motor skills and more highly stimulated senses, which means that they have more capacity to focus on the surroundings, the social relationships or the cognitive challenges that they face when they play and learn.

#### All children must have an elevated pulse every day

A normal day in daycare contains a variety of activities that strengthen various bodily skills. At the same time, we know that there is a correlation between the intensity of physical activity one develops and its health effects, and that physical activity of high intensity has the greatest effects. Therefore, all children in the Gazelle Children’s Houses and Gazelle Childcare Centres must have an elevated pulse every day.

- 1. **The individual daycare centre must develop learning environments in accordance with the *Learning Environments in Active Children in Daycare*, which is published as an appendix to this concept.**

The decor of the learning environments through which children move greatly affects their behaviour. Therefore, it is important that learning environments are created where the employees and the decor are conducive to physical activity, and where physical activity is recognized as something positive and used as an educational tool.

#### The employees' role and training

Children's natural behaviour is largely determined by whatever gives them pleasure. Therefore, as a rule, children play the games they want. This means that the daycare centre employees have a task in creating motivation amongst children so that they have a natural desire to be physically active. It is all about introducing games and activities that create joy and give children a sense of mastery. There must be a culture where there is room to experiment and practise. This applies to both children and adults.

Employees must challenge their own physicality and lead the way as role models who will, can and dare.

At the same time, centre employees have the task of involving the body and movement as an educational tool and of developing activities that address and involve all children.

All employees have to undergo a 37-hour competency development program with a view to making this concept work in reality. The competency development program will be described in a separate document which will, amongst other things, contain knowledge about children's physical and sensory-motor development, activity development, motivation and the use of physical activity as a pedagogical tool.

#### Parental Involvement

The parents are kept informed about the project on a current basis, and a separate description of the project is prepared that targets the parents. Each Gazelle Children’s House and the individual Gazelle Childcare Centre handles the concrete and practical information on the project, and creates a continuous and constructive dialogue with the parents about the child's well-being, development, learning and upbringing.

#### Organization

*The project is an interdisciplinary project between the discipline areas of Children and Youth, Social and Health, Culture, Business and Development. Children and Youth handle the coordination of the project.

*The project is politically anchored in the Committee for Children and Youth, the Social and Health Committee and the Business, Employment and Culture Committee.

*Each Gazelle Children’s House and Childcare Centre appoints coordinators who participate in a network and function as contact people.

*The teaching team leaders in the Gazelle Children’s Houses and Childcare Centres participate in a specially organized competency development program

*The teaching team leaders in the Gazelle Children’s Houses and Childcare Centres must implement a process in the employee group that relates to preparing a script, which as a minimum:

- - - describes how the concept is implemented in each Gazelle Children’s House and individual Gazelle Childcare Centre
    - describes how the five Gazelle leaps are implemented. A working example of a weekly schedule is prepared
    - describes how parents are involved in and informed about the project.

#### Sharing of knowledge

The designated coordinators can be both managers and employees. Coordinators participate in regular networking meetings where ideas are shared and the latest information on the project is provided. During the project start-up, the network meets every second month. Besides the network, an idea bank is created where employees can find and share inspiration for activity development and creative ideas. There must be opportunities to share pictures and videos.

Gazelle Children’s Houses are also encouraged to occasionally utilize each other's employees in order to exploit special skills and interests, and in this way, create an even more varied offering of activities for the children.

In addition, it is recommended that daycare centres make joint investments in portable tools that can rotate between the different centres and children's houses.

#### Research and evaluation

Like the sports school project in The Svendborg Project, it is also expected in *Active Children in Daycare* that a research collaboration will be established with the University of Southern Denmark, which can follow the development and measure the effect of the project. Unfortunately, there are no usable methods for 0-2- year old children, which is why the research includes only preschool children.

It will, of course, be voluntary for parents if they want their children to participate in the research. Parents receive, in due time, comprehensive information on the research and testing, so they can make an informed choice.

#### Knowledge, experience and environment

According to the Danish Health and Medicines Authority, children with the worst fitness, have about 15 times greater risk of accumulating the risk factors associated with lifestyle diseases. At the same time, the Authority points out that there is a widening gap between children with the worst fitness and children with the best fitness. There is no doubt that physical activity promotes health and prevents many diseases. As far as children are concerned, the risk of lifestyle diseases such as cardiovascular disease, obesity and diabetes is reduced by increasing physical activity. Therefore, it is important that efforts are targeted to *all* children.

The Svendborg Project has confirmed that increased physical activity promotes children's health. The research has thus documented that:

- The children in the sports schools have significantly lower risk of developing lifestyle diseases such as cardiovascular diseases and type II diabetes
- The children in sports schools are in better physical shape, stronger and less obese
- The children in sports schools have better motor skills
- The children in sports schools have lower risk of back problems
- The children who most need a health boost benefit most from the additional sports
- Mathematics teaching with physical activity enhances learning by 35% compared to that involving sedentary learning

Schools also report that there are fewer conflicts and the children are happier and more able to concentrate.

Physical activity is defined in this context as "any movement that increases energy yield, ie both unstructured activity and more conscious, purposeful, regular physical activity". Physical activity may also have different intensity. Moderate physical activity includes all forms of activity / exercise where the heart rate increases but where one can still talk with others. Physical activity of high intensity can be scheduled training / physical activity, where the heart rate increases, so one feels breathless and finds it difficult to hold a conversation.

When we talk about health in this context, health as a broad concept is considered. In accordance with the Svendborg Municipality’s health policy, health is defined in this project as: *"Health is a condition of complete physical, mental and social well-being and not merely absence of disease and infirmity ".* Health is therefore about much more than physical and bodily well-being, and physical activity is an effective tool to work with all these elements.

Physical activity and motor development is therefore something that is already being worked on in day care, where, by law, it has its own educational curriculum theme, *Body and movement,* which, in the guide, is described as follows:

*"Children develop basic motor skills in childhood, which enhance their ability to develop. Daycare should contribute to strengthening children's development of motor skills, stamina and mobility. Daycare uses nature and immediate surroundings as opportunities for physical activities and challenges. By actively exploring the body's possibilities and limitations, children's skills and habits develop. They get experience in what it means to connec,t for example, spoken language and body language, and thus they also develop respect for other people's expressions and reactions. "*

It is this knowledge, these experiences and these frameworks from which *Active Children in Daycare* should be viewed.

#### Sources and inspiration

Danmarks Idrætsforbund / KFUMs Idrætsforbund: *Rend og hop med Oliver*

Grethe Sandholm, 2012: *Pædagogen som kropslig kulturformidler via pædagogisk idræt*

Heller Stegger & Hannah Harboe, 2013: *Pædiatrisk Fysioterapi* Sundhedsstyrelsen, 2011: *Håndbog for fysisk aktivitet* Sundhedsstyrelsen, 2012: *Forebyggelsespakke – Fysisk aktivitet*

Team Danmark: *Aldersrelateret træning – Målrettet og forsvarlig træning af børn og unge*

WHO: *Constitution of The World Health Organization*.

Ylva Ellneby, 2000: *Om børn og stress – og hvad vi kan gøre ved det*
